# Genotypic Variation of Nitrogen Use Efficiency and Amino Acid Metabolism in Barley

**DOI:** 10.3389/fpls.2021.807798

**Published:** 2022-02-04

**Authors:** Bérengère Decouard, Marlène Bailly, Martine Rigault, Anne Marmagne, Mustapha Arkoun, Fabienne Soulay, José Caïus, Christine Paysant-Le Roux, Said Louahlia, Cédric Jacquard, Qassim Esmaeel, Fabien Chardon, Céline Masclaux-Daubresse, Alia Dellagi

**Affiliations:** ^1^Université Paris-Saclay, INRAE, AgroParisTech, Institut Jean-Pierre Bourgin (IJPB), Versailles, France; ^2^Agro Innovation International - Laboratoire Nutrition Végétale, TIMAC AGRO International SAS, Saint Malo, France; ^3^Université Paris-Saclay, CNRS, INRAE, University of Évry Val d′Essonne, Institute of Plant Sciences Paris-Saclay (IPS2), Orsay, France; ^4^Université de Paris, CNRS, INRAE, Institute of Plant Sciences Paris-Saclay (IPS2), Orsay, France; ^5^Natural Resources and Environment Lab, Faculté Polydiscipliniare de Taza, Université Sidi Mohamed Ben Abdellah, Taza, Morocco; ^6^Université de Reims Champagne Ardenne, RIBP EA 4707 USC INRAE 1488, SFR Condorcet FR CNRS 3417, Reims, France

**Keywords:** NUE (nitrogen use efficiency), crop/stress physiology, barley, natural variability, lysine (amino acids)

## Abstract

Owing to the large genetic diversity of barley and its resilience under harsh environments, this crop is of great value for agroecological transition and the need for reduction of nitrogen (N) fertilizers inputs. In the present work, we investigated the diversity of a North African barley genotype collection in terms of growth under limiting N (LN) or ample N (HN) supply and in terms of physiological traits including amino acid content in young seedlings. We identified a Moroccan variety, Laanaceur, accumulating five times more lysine in its leaves than the others under both N nutritional regimes. Physiological characterization of the barley collection showed the genetic diversity of barley adaptation strategies to LN and highlighted a genotype x environment interaction. In all genotypes, N limitation resulted in global biomass reduction, an increase in C concentration, and a higher resource allocation to the roots, indicating that this organ undergoes important adaptive metabolic activity. The most important diversity concerned leaf nitrogen use efficiency (LNUE), root nitrogen use efficiency (RNUE), root nitrogen uptake efficiency (RNUpE), and leaf nitrogen uptake efficiency (LNUpE). Using LNUE as a target trait reflecting barley capacity to deal with N limitation, this trait was positively correlated with plant nitrogen uptake efficiency (PNUpE) and RNUpE. Based on the LNUE trait, we determined three classes showing high, moderate, or low tolerance to N limitation. The transcriptomic approach showed that signaling, ionic transport, immunity, and stress response were the major functions affected by N supply. A candidate gene encoding the HvNRT2.10 transporter was commonly up-regulated under LN in the three barley genotypes investigated. Genes encoding key enzymes required for lysine biosynthesis in plants, dihydrodipicolinate synthase (DHPS) and the catabolic enzyme, the bifunctional Lys-ketoglutarate reductase/saccharopine dehydrogenase are up-regulated in Laanaceur and likely account for a hyperaccumulation of lysine in this genotype. Our work provides key physiological markers of North African barley response to low N availability in the early developmental stages.

## Introduction

Barley is a staple crop known for its great adaptability to harsh environments. It was one of the first domesticated crops and is the fourth most productive cereal crop after rice, wheat, and maize (FAOSTAT). Barley (*Hordeum vulgare* L.) shows a very large genetic diversity and is grown under a large array of environmental and soil conditions with areas of production at high altitudes and latitudes as well as in desert regions (Ryan and Sommer, 2012; Muñoz-Amatriaín et al., 2014; Dawson et al., 2015).

Barley is mainly used for animal feed, human consumption, and malting. Today, barley is gaining value in the field of nutrition, not only for its original flavor but also for its nutritional value especially because of its high content in β-glucans and low gluten (Baik and Ullrich, 2008; Chutimanitsakun et al., 2013). Barley is considered for several benefits to human health, such as reduction of blood cholesterol and glucose levels as well as weight loss by increased satiety, control of heart disease, and type-2 diabetes (Baik and Ullrich, 2008). In some parts of the world, such as Ethiopia, North Africa, and Asia, it is used in human food more frequently than in the rest of the world (Baik and Ullrich, 2008).

Mediterranean climate and soils impose drastic constraints on agriculture. Barley is one of the best-adapted species to the Mediterranean conditions (Pswarayi et al., 2008). Climate change and the growing Mediterranean population will further increase environmental constraints on barley culture in a near future (Cammarano et al., 2019). Fortunately, barley shows great potential for biomass production under Mediterranean climates. As is the case for most cereals, barley yields are strongly dependent on nitrogen fertilization (Oscarsson et al., 1998; Sedlář et al., 2011; Stupar et al., 2017). Importantly, nitrogen fertilization impacts plant tolerance to abiotic and biotic stresses (Fagard et al., 2014; Abid et al., 2016; Mur et al., 2017; Ding et al., 2018; Verly et al., 2020). The genetic diversity in terms of barley tolerance to nitrogen starvation has been explored (Oscarsson et al., 1998; Górny, 2001; Sinebo et al., 2004; Quan et al., 2016, 2019; Karunarathne et al., 2020). However few data are available concerning the diversity of molecular responses of barley to nitrogen limitation (Møller et al., 2011; Quan et al., 2016, 2019; Karunarathne et al., 2020, 2021).

World agriculture benefited from unprecedented changes in agronomic practices during the “Green Revolution” due to technological progress after Second World War. Major crop yields doubled per capita over a 50 year period in some regions of the world, such as Asia and South America (Lassaletta et al., 2016; Pretty, 2018). During that period, new crop varieties were bred, and inorganic fertilizers and chemically synthesized pesticides and herbicides were produced and used. Their application was combined with the modernization of agricultural machinery (Lassaletta et al., 2016; Pretty, 2018). In particular, it is estimated that the use of synthetic inorganic nitrogen (N) fertilizers has increased 8- during the last 50 years (Lassaletta et al., 2016; Pretty, 2018). Nowadays, the industrial Haber-Bosch process uses 1–2% of the world’s fossil-fuel energy output for the synthesis of ammonia that is the basis for the production of the other N fertilizers as nitrate salts, ammonium-nitrate, and urea (Chen et al., 2018). However, because crops do not take up more than 30–50% of the N available in the soil (Wang et al., 2018), the extensive use of N fertilizers caused major detriments to ecosystems and animal health (Schlesinger, 2009; Withers et al., 2014).

In the context of a growing population and shrinking farmlands, cereals yields and nutritional quality is fundamental because cereal grains provide 60% of the food necessary to feed the world population, either directly as part of the human diet or indirectly as animal feed (Hirel et al., 2007; Lafiandra et al., 2014; Landberg et al., 2019). Nitrogen is one of the key elements that determine plant growth and yield formation (Hirel et al., 2007; Masclaux-Daubresse et al., 2010). It is thus essential to optimize N use efficiency (NUE) in crops. NUE is most commonly defined as the grain or biomass yields obtained per unit of available N in the soil (Xu et al., 2012; Han et al., 2015; Li et al., 2017; Hawkesford and Griffiths, 2019). Nitrogen uptake refers to processes involved in the acquisition of nitrogen compounds from the soil. Nitrogen assimilation refers to processes associated with the N utilization and N metabolism that transform inorganic nitrogen into organic nitrogen *in planta*. Nitrogen remobilization refers to processes associated with the recycling and reuse of organic nitrogen within the plant and its transfer from organs to organs. Nitrogen uptake, assimilation, and remobilization contribute to plant NUE (Hirel et al., 2007; Lea and Miflin, 2018) that can be also estimated considering the three components that are N uptake efficiency (NupE), N utilization efficiency (NutE), and nitrogen remobilization efficiency (NRE) (Han et al., 2015; Li et al., 2017).

Nitrogen (N) is present in the soil in the form of nitrate (NO_3_^–^), ammonium (NH_4_^+^), or amino acids, with their availability depending upon physical factors, such as pH and temperature. Most plants adapted to alkaline pH in aerobic soils, which is the case for most arable lands, use mostly NO_3_^–^ as their N source (Hirel et al., 2007; Masclaux-Daubresse et al., 2010; O’Brien et al., 2016; Xu, 2018). Nitrate is taken up by the roots and then transported in the plant via plasma membrane located transporters that are either low-affinity transporters (LATs) or high-affinity transporters (HATs) (Léran et al., 2014; O’Brien et al., 2016; Kant, 2018; Wang et al., 2018; Zhang et al., 2018). Following uptake, NO_3_^–^ is reduced to nitrite (NO_2_^–^) by the cytosolic enzyme nitrate reductase (NR). Then, NO_2_^–^ is further reduced to ammonium by the plastid nitrite reductase (NiR). Ammonium derived from direct uptake or NO_3_^–^ reduction is finally incorporated into amino acids *via* the combined activity of the two enzymes glutamine synthase (GS) and glutamate synthase (GOGAT) (Masclaux-Daubresse et al., 2010; Wang et al., 2018; Hirel and Krapp, 2020).

Although barley is a major crop requiring N fertilization in poor soils, such as those of North Africa, and despite functional and evolutionary genomics tools developed on this species, little is known about the diversity of physiological and molecular mechanisms in barley responses to N limitation.

In the present work, we investigated the diversity of a collection of north African barley genotypes in terms of growth under limiting N conditions and in terms of N nutrition physiological traits related to N nutrition including amino acid content that led to the identification of a barley genotype accumulating five times more lysine than the others. To gain further insight into the molecular mechanisms involved in barley adaptation to N limitation, a transcriptomics approach revealed that N supply has an impact on ionic transport, signaling, stress responses, and immunity. We identified candidate genes controlling N deficiency response and lysine biosynthesis in barley.

## Materials and Methods

### Plant Material and Growth Conditions

The origin of barley genotypes is indicated in Table 1. Seeds were provided by M. Bennaceur from the National Gene Bank of Tunisia and by Université Sidi Mohamed Ben Abdellah. The barley North African collection used in this study contains nine Moroccan genotypes that correspond to commercialized varieties (herein named M1 to M9), one Tunisian variety (herein named T6), and one Egyptian variety (herein named E6) Table 1. The North African barley collection used in this study displays different characteristics in particular, with regard to their yield and tolerance to drought (Hellal et al., 2019) and it was recently described for its response to Cd (Ayachi et al., 2021). The European cultivar Golden Promise (herein named GP), which is a reference genotype since its genome is fully sequenced and for which Agrobacterium-mediated transformation is possible (Schreiber et al., 2020), was included in the analyses as a reference line. Seeds were surface-sterilized then sown on the sand under long days 16 h day (23°C)/8 h night (18°C). They were watered three times a week with the same nutrient solution containing either 0.5 mM nitrate (Low N, LN) or 5 mM ample nitrate (High N, HN). Reducing tenfold nitrate concentration involves necessarily compensation of counterion changes. Although this is not a perfect method, there is no other way for that and most care was taken to design the mineral composition of the nutritional solution so that there is no other major deficiency or toxicity. Most importantly, the K levels are not limiting both under LN and HN (Epstein et al., 1963; Gierth and Mäser, 2007; Genies et al., 2021). Watering was applied by sub-irrigation of the pots and maintained for 2 h before nutritive solutions were discarded. The nutrient solution composition is described in Supplementary Table 1. Plants were harvested 14 days after sowing by separating shoot and root which were weighed separately. The experiments were performed four times with eight plants in each biological replicate.

**TABLE 1:** Names and characteristics of the barley collection genotypes used in this study.

Code in this work	Official name	Country of origin	References describing the genotype	Row type	Spring/winter type	Hulled/hulless	Earliness of maturity (Badraoui et al., 2009; Noaman et al., 2007; Mlaouhi et al., 2020; Saidi et al., 2005)	Disease resistance (Badraoui et al., 2009; Noaman et al., 2007; Saidi et al., 2005)	Year of release
M1	Adrar	Morocco	Hellal et al., 2019	2 rows	Spring type	Hulled	Medium type	Resistant to powdery mildew, susceptible to Rhynchosporium, moderately resistant to rust	1998
M2	Taffa	Morocco	Hellal et al., 2019	6 rows	Winter type	Hulled	Medium type	Moderately resistant to powder mildew and rust; susceptible to Rhynchosporium	1994
M3	Massine	Morocco	Hellal et al., 2019	6 rows	Winter type	Hulled	Medium type	Moderately resistant to powdery mildew and yellow rust, susceptible to Rhynchosporium and moderately susceptible to brown rust	1994
M4	Laannaceur	Morocco	Hellal et al., 2019	6 rows	Winter type	Hulled	Medium type	Moderately susceptible to powdery mildew and Rhynchosporium, susceptible to rust	1991
M5	Oussama	Morocco	Hellal et al., 2019	6 rows	Winter type	Hulled	Medium type	Susceptible to powdery mildew and Rhynchosporium, susceptible to yellow and brown rust	1995
M6	Firdaws	Morocco	Hellal et al., 2019	6 rows	Winter type	Hulled	Medium type	Resistant to powdery mildew	1998
M7	Tamellalt	Morocco	Hellal et al., 2019	2 rows	Spring type	Hulled	Medium type	Moderately susceptible to powdery mildew, susceptible to Rhynchosporium, moderately resistant to yellow and brown rust	1984
M8	Amalou	Morocco	Hellal et al., 2019	6 rows	Winter type	Hulled	Early type	Moderately resistant to powdery mildew, susceptible to Rhynchosporium, moderately resistant to yellow and brown rust	1997
M9	Amira	Morocco	Hellal et al., 2019	6 rows	Winter type	Hulled	Medium type	Resistant to powdery mildew, susceptible to Rhynchosporium and rust	1996
T6	Manel	Tunisia	Ben Naceur et al., 2012	6 rows	Spring type	Hulled	Early type	Moderately resistant to powdery mildew and Rhynchosporium, moderately resistant to net blotch	1996
E6	Giza 2000	Egypt	Ben Naceur et al., 2012	6 rows	Spring type	Hulled	Early type	Moderately resistant to leaf Rust. Resistant to powdery mildew and net blotch	2003
GP	Golden Promise	Europe	Avila-Ospina et al., 2015	2 rows	Spring type	Hulled	Early type	Susceptible to net blotch and powdery mildew	1968

### Determination of Nitrate Uptake Into the Shoots and Roots Using ^15^N Labeling During 24 h

To determine ^15^N uptake over 24 h before harvesting, thus on day 13 after sowing, a ^15^N labeling was performed. On day 13 after sowing, the unlabeled watering solution was replaced by a ^15^N-containing solution that had the same nutrient composition as the Low N and High N solutions except that the natural ^14^NO_3_^–^ was replaced by nitrate with 10% ^15^NO_3_^–^ enrichment (w/w). All the pots were watered for 24 h, using an equal volume of labeled solutions. Cutting the shoots stopped ^15^N uptake in the shoots. Roots were extracted from sand and carefully rinsed before freezing in liquid nitrogen. Shoot and root tissues were harvested, weighed for fresh weight quantification, then ground in liquid nitrogen and stored at -80°C for further experiments.

Dry weight was calculated based on the weight of lyophilized tissues for amino acid analysis (see below). This allowed the calculation of the percentage of dry matter in each sample.

### Quantification of Total Nitrogen, Total Carbon, and ^15^N Enrichment

The experiment dried again 50 mg of ground frozen plant material before weighting 5,000 μg of dry material in tin capsules to determine the total N and C concentrations using the FLASH 2000 Organic Elemental Analyzer (Thermo Fisher Scientific Villebon, France) and the ^15^N enrichment using the Delta V Advantage isotope ratio mass spectrometer (Thermo Fisher Scientific, France). The data obtained are N% (g of N per 100 g of DW), C% (g of C per 100 g of DW), and A% (Atom percent) that represent the^ 15^N enrichment in the sample [^15^N/(total N)]. Since the natural ^15^N abundance in N labeled samples was 0.3663 (A% control), specific enrichments due to the ^15^N uptake were calculated as E% = (A% - 0.3363).

### Amino Acid Analysis

For amino acid determination, 10 mg of lyophilized dry matter was extracted with a solution containing 400 μl of MeOH and 0.25 nmol/μl of Norvaline, which was used as the internal standard (Sigma Aldrich, St. Louis, MO, United States). The extract was stirred for 15 min, and it was then re-suspended with 200 μl of chloroform (agitation for 5 min) and 400 μl of double-distilled water (ddH_2_O). After centrifugation (12,000 rpm, 10°C, 5 min), the supernatant was recovered, evaporated, and dissolved in 100 μl of ddH_2_O. Derivatization was performed using an Ultra Derivatization Kit AccQ tag (Waters Corp, Milford, MA, United States), following the protocol of the manufacturer (Waters Corp, Milford, MA, United States). The amino acid profile was determined by ultra-performance liquid chromatography coupled with a photodiode array detector (UPLC/PDA) H-Class system (Waters Corp, Milford, MA, United States) with an ethylene bridge hybrid (BEH) C18 100 × 2.1 mm column (pore size: 1.7 μm).

### Plant Growth and N Nutrition Trait Indicators

The plant phenotypic traits and indicators were measured or calculated based on the formula detailed in Supplementary Table 2.

### Inoculum Preparation and Pathogen Infection

For each of the three barley cultivars M4, M5, and GP, seeds were sown at a rate of 10 seeds/pot in plastic pots 7 cm in diameter filled with 300 g of sand. They were watered three times a week with either LN or HN solutions. Plants were grown in a growth chamber (Aralab) at 23°C under white fluorescent light (130 μmol m^–2^ s^–1^), with a 14- and 10-h photoperiod and 80% relative humidity as previously described by Backes et al. (2021b). A detached leave assay was carried out to evaluate the susceptibility of the three genotypes to the pathogen *Pyrenophora teres*. For each condition, 30 plants were inoculated and recorded. Three independent biological replicates were performed. Briefly, leaves of 10-day-old barley plants were excised and placed on Petri dishes containing 1% of agar. Leaves were then injured with a wooden pick and then a volume of 10 μl of suspensions containing *P. teres* spores at a concentration of 10^5^ spores/mL was deposited at the leaf wound area. The incidences of net blotch disease symptoms, represented by the presence of necrosis on barley leaves, were recorded at 10 days post-infection.

### RNA-Seq Analysis

Furthermore, three independent biological replicates were produced. Leaves were collected on plants at three leaf developmental growth stages corresponding to 14 days after sowing, cultivated in two conditions, LN or HN. Each sample is composed of the leaf (tissue) of 1–2 plants. Total RNA was extracted using the Nucleosol extraction kit according to the supplier’s instructions and was further purified using the RNA Clean & Concentrator Kits (Zymo Research^®^, California, United States). RNA-seq libraries were constructed by the POPS platform (IPS2) using the TruSeq Stranded mRNA library prep kit (Illumina^®^, California, United States) according to the supplier’s instructions. The libraries were sequenced in Single end (SE) mode with 75 bases for each read on a NextSeq500 to generate between 5 and 62 million SE reads per sample.

Adapter sequences and bases with a Q-Score below 20 were trimmed out from reads using Trimmomatic (version 0.36; Bolger et al., 2014) and reads shorter than 30 bases after trimming were discarded. Reads corresponding to rRNA sequences were removed using sortMeRNA (version 2.1; Kopylova et al., 2012) against the silva-bac-16s-id90, silva-bac-23s-id98, silva-euk-18s-id95, and silva-euk-28s-id98 databases.

Filtered reads were then mapped and counted using STAR (version 2.7.3a; Dobin et al., 2013) with the following parameters –alignIntronMin 5 –alignIntronMax 60000 –outSAMprimaryFlag AllBestScore –outFilterMultimapScoreRange 0 –outFilterMultimapNmax 20 on the Hordeum_vulgare.IBSC_v2.48.gtf and its associated GTF annotation file.

Between 76.28 and 77.7% of the reads were associated with annotated genes (a mean of 76.9, 76.6, and 76.7%, respectively for GP, M4, and M5 barley cultivars). Statistical analysis was performed with Wilcoxon’s test (Supplementary Figure 7). When comparing the percentages of assigned read samples per cultivar, the difference between the means is not statistically significant. The three cultivars mapped similarly onto Morex reference. Morex reference has a higher version (v2) than GP reference (v1). The reference annotation should be better for Morex.

A gene is analyzed if it is present at more than 1 read per million in several samples greater than or equal to the minimum number of replicates. The resulting raw count matrix was fed into edgeR (Robinson et al., 2010) for differential expression testing by fitting a negative binomial generalized log-linear model (GLM) including a condition factor and a replicate factor to the TMM-normalized read counts for each gene. We performed pairwise comparisons of each of the depleted conditions to the control condition. The distribution of the resulting *p*-values followed the quality criterion described by Rigaill et al. (2018). Genes with an adjusted *p*-value (FDR; Benjamini and Hochberg, 1995) below 0.05 were considered as differentially expressed.

### Statistical Analysis

Analysis of variance followed by Tukey’s honestly significant difference (HSD) test, as well as two-sample *t*-tests, were used in this study. All statistical analyses were performed using the free software environment R Version 4.0.2.1. The least-square means were calculated using the R package emmeans.

## Results

### Global Trends of the Impact of Nitrogen Nutrition on Barley Physiological Traits

Although barley is commonly grown in North Africa, little is known about the mechanisms involved in its tolerance to low N availability, a common feature in this cultivation area. Global changes for N nutrition physiological indicators in the barley species were determined depending on nitrogen availability by considering the entire barley collection (Table 2). Nitrogen limitation resulted in the reduction of plant DW mainly due to a decrease in leaf DW. By contrast, root DW was higher under LN compared to HN, which globally resulted in a decrease of the shoot/root ratio (SR) under LN. As expected, barley nitrogen concentration was strongly reduced under LN irrespective of a plant organ. In contrast, carbon concentration was higher under LN. As expected, the global trend of the collection indicates that nitrogen uptake efficiencies (LNUpE, RNUpE, and PNUpE) were lower under LN in both shoots and roots, certainly due to the fact that nitrate was less available under LN. The biomass produced per unit of N reflects nitrogen use efficiency (NUE) in plants at the vegetative stage. As such NUE, in our case, can be calculated as the ratio between biomass and N concentration (Chardon et al., 2010). As expected, NUE was higher under LN than under HN. It is interesting to notice that leaf NUE (LNUE) was slightly lower under N limitation than under HN, while root NUE (RNUE) was sharply higher under LN than HN (Table 2). Partitioning of dry matter and N was different under LN and HN. Dry matter and N partitioning in roots, RP%DW and RP%N, respectively, were higher under LN than under HN (Table 2), thus reflecting the fact that shoot/root was decreased under LN relative to HN. Similarly, under LN, nitrogen was taken up more efficiently to the roots than to the shoots. This is illustrated by the higher partitioning of ^15^N in roots (RP%15N) under LN compared to HN (Table 2).

**TABLE 2:** Comparison of global trends of physiological traits within the barley collection under HN and LN.

Short-name	Trait name and unit	HN	*> or *<	LN
PDW	Plant dry weight (mg/plant)	78.18	ns	59.47
LDW	Leaf dry weight (mg/plant)	58.57	*>	39.56
RDW	Root dry weight (mg/plant)	19.07	*<	19.82
PN%	Nitrogen concentration in the whole plant (gN/100 gDW)	5.17	*>	3.32
LN%	Nitrogen concentration in shoot (gN/100 gDW)	5.67	*>	3.83
RN%	Nitrogen concentration in root (gN/100 gDW)	3.69	*>	2.32
PC%	Carbon concentration in the whole plant (gC/100 gDW)	35.2	*<	37.19
LC%	Carbon concentration in shoot (gC/100 gDW)	35.92	*<	37.85
RC%	Carbon concentration in root (gC/100 gDW)	32.99	*<	36.28
PNUE	Plant NUE (mg DW/%N)	15.3	*<	16.9
LNUE	Leaf NUE (mg DW/%N)	10.72	*>	9.92
RNUE	Root NUE (mg DW/%N)	5.38	*<	7.33
PNUpE	Plant N uptake efficiency mg ^15^N/100 mg DW	4.11	*>	3.63
LNUpE	Leaf N uptake efficiency mg ^15^N/100 mg DW	3.77	*>	3.26
RNUpE	Root N uptake efficiency mg ^15^N/100 mg DW	4.92	*>	4.46
LP%DW	Biomass partitioning in shoot	0.75	*>	0.67
RP%DW	Biomass partitioning in root	0.25	*<	0.33
SR	Shoot DW to root DW ratio	3.08	*>	1.96
LP%N	Nitrogen partitioning in shoot	0.83	*>	0.75
RP%N	Nitrogen partitioning in root	0.17	*<	0.25
LP%15N	^15^N partitioning in shoot	0.7	*>	0.6
RP%15N	^15^N partitioning in root	0.3	*<	0.4
PNUp	Plant N uptake (Total _Nitrogen.Uptake) (mg ^15^N per plant/h)	4.11	*>	3.62

HN and LN indicate the mean of the considered trait over the whole individuals of the collection under high or low N, respectively. *> or *< indicates that the mean is significantly different between HN and LN, t student p < 0.05. SE, standard error for the variable over the whole individuals of the collection; ns, non-significant.

Altogether these data indicate that barley responds to nitrogen limitation by a global biomass reduction, an increase in C concentration, and a higher resource allocation (DW, N, and C) to the roots.

### Exploring Natural Variation for N and C Management Within the Barley Collection

To determine the effect of genotype (G) and N nutrition on the nitrogen-related physiological indicators, a three-way ANOVA was applied. We also checked the interaction between these factors (GXN) and the effect of biological replicates (E). ANOVA results in concerned traits related to (i) biomass and elemental (C,N) distribution in aerial parts and roots presented in Table 3A; (ii) plant capacity to take up and use N for biomass production (Table 3B); (iii) relative partitioning of biomass, C and N between leaves and roots (Table 3C).

**TABLE 3:** Level of significance of the variance sources for biomass and C and N concentrations in barley cultivated under limiting or ample nitrate.

A:	Level of significance												Sum of squares					
	L DW	R DW	P DW	LN%	RN%	PN%	LC%	RC%	PC%	L DW	R DW	P DW	LN%	RN%	PN%	LC%	RC%	PC%
G	***	***	***	***	**	***	***	***	***	25.0	29.1	23.4	2.1	3.0	1.0	3.8	9.0	6.5
N	***		***	***	***	***	***	***	***	**40.9**	0.6	29.1	**82.4**	**58.7**	**76.7**	**77.5**	29.5	**50.2**
E	***	***	***	***	***	***	***	***	***	7.9	8.4	8.5	5.5	12.0	10.7	1.5	6.4	6.4
GXN	***	***	***	***		***	***	*		8.0	13.4	3.8	2.1	1.2	1.1	3.0	5.0	1.6
GXE	**		***	**		***	***		**	4.6	10.5	7.7	1.4	3.8	1.9	6.2	7.0	8.6
NXE			***	***	***	***		***	***	0.3	0.8	1.8	2.6	2.9	4.1	0.1	4.3	4.7
GXNXE	*		***	*		*				3.5	6.6	7.4	1.0	2.1	1.0	1.0	5.9	3.1
R										9.8	**30.6**	18.2	2.9	16.4	3.4	6.9	**32.9**	18.9

B:	Level of significance							Sum of squares						
	LNUE	RNUE	PNUE	LNUpE	RNUpE	PNUpE	PNUp	LNUE	RNUE	PNUE	LNUpE	RNUpE	PNUpE	PNUp
G	***	***	***	***	***	***	***	**40.0**	14.1	**33.2**	15.1	14.8	12.8	**23.7**
N	***	***	***	***	***	***	***	2.6	12.0	5.8	9.1	3.8	3.5	23.1
E	***	***	***	***	***	***	***	2.7	19.0	4.2	20.9	21.5	**32.7**	14.5
GXN	***		**	**	**		**	12.6	3.5	6.1	4.9	7.3	3.0	3.0
GXE	**	*	*	**			**	8.7	9.7	10.1	10.1	4.6	5.4	6.5
NXE	*	***	**	***	***	***	***	1.4	4.4	2.5	8.3	7.0	9.7	4.2
GXNXE	***	*	**	**			***	11.4	9.3	10.3	9.5	8.7	7.1	8.3
R								20.6	**28.0**	27.8	**22.0**	**32.4**	25.8	16.6

C:	Level of significance							Sum of squares						
	LP%DW	RP%DW	SR	LP%N	RP%N	LP%15N	RP%15N	LP%DW	RP%DW	SR	LP%N	RP%N	LP%15N	RP%15N
G	**	***		***	***	***	***	5.0	6.1	2.1	5.4	5.8	7.0	6.8
N	***	***	***	***	***	***	***	**32.1**	**37.1**	**41.9**	**51.9**	**52.2**	**50.4**	**54.0**
E	***	***	***	***	***	***	***	14.9	15.8	24.1	8.4	8.7	4.4	3.5
GXN	**	***	***	***	***	***	***	5.3	6.0	3.9	4.7	4.6	6.0	6.6
GXE	*							9.0	5.6	4.7	5.9	6.1	4.7	4.3
NXE		**		***	***	**	**	1.2	1.7	0.8	2.6	2.2	1.9	2.1
GXNXE	*	*						6.7	6.1	5.1	2.7	3.0	3.0	3.2
R								25.8	21.5	17.5	18.3	17.4	22.5	19.5

*G, N, and E stand for the following factors respectively: “Barley genotype,” “N nutrition,” and “experiment replicate.” The significance of the interaction between these factors is indicated as follows GXN, NXE, GXE, and GXNXE. R: residuals. The highest sum of squares for each trait is in bold. Three-way ANOVA was applied to the data set. Sub-tables represent A: biomasses, C and N content; B: Nitrogen uptake and use efficiency, C: partitioning of biomass, C and N.*

*Significant at 0.05 probability level.

**Significant at 0.01 probability level.

***Significant at 0.001 probability level.

These data show that nitrate nutrition significantly affects all traits except root dry weight.

The genotypic effect was also significant for most physiological traits, except the shoot/root (SR) ratio. Interestingly, the N-uptake (PNUp) and NUE (LNUE and PNUE) related traits are more impacted by the genotype effect than by the nutrition effect, as indicated by the sum of squares for the genotype effect which is larger than that of the nutrition effect (Table 3). Thus, in barley, NUE can be improved via breeding since the genetic factor plays a significant role in this trait.

**FIGURE 1:**
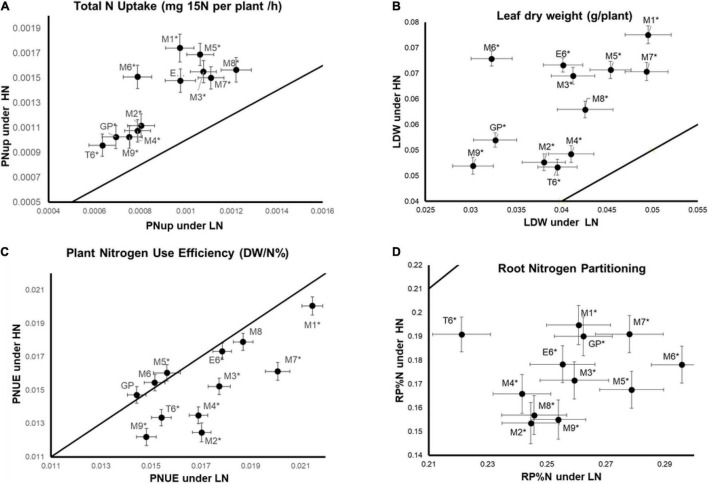
Different genotypes by N supply interactions within the barley North African collection. Plants were grown for 14 days under LN or HN then leaves and roots were harvested separately and frozen under liquid nitrogen. The physiological parameters were measured as indicated in the “Materials and Methods” section. Traits displaying GXN interactions are illustrated by different GXN patterns. **(A)** Total plant N uptake (PNUpE). **(B)** Leaf dry weight (LDW). **(C)** Plant nitrogen use efficiency (PNUE). **(D)** Root nitrogen partitioning (RP%N). Mean values under HN are plotted against mean values under LN. Four independent experiments were performed. Stars indicate a significant difference between LN and HN (Student’s test, 13 ≤ *n* ≤ 16, *p* < 0.05). Bars represent SE.

The genotypic by nutrition (GxN) interaction effect is significant for several traits for which the plant response to the nutrition depends on the genotype (Table 3). For example, the total N uptake (PNUp) is higher under HN than LN for all genotypes and we can clearly distinguish two groups of genotypes with different PNUp under HN and LN. The GP, M2, M4, M9, T6 genotypes exhibit the lowest PNUp scores whereas the PNUp of M1, M3, M5, M6, M7, M8, and E6 reached higher scores (Figure 1A). The clustering of these genotypes follows the same trend for leaf DW (LDW) but only under HN (Figure 1B) indicating that these traits are correlated as shown in Supplementary Figure 1. For plant NUE (PNUE), it is not possible to cluster genotypes in different groups. We can notice five genotypes (GP, M6, M8), showing similar PNUE values under HN and LN, while all the others present lower PNUE under HN than LN (Figure 1C). This suggests that for the five genotypes mentioned above, low N does not affect NUE. Last, for root N partitioning (RP%N), all genotypes show lower values under HN than under LN, but the T6 genotype clearly behaves as an outlier with significantly lower RP%N under LN compared to other accessions (Figure 1D).

These four examples illustrate the diversity of the pattern of GxN responses in the barley collection. Thus, depending on the trait we observe, nutrition may cause different modifications according to the genotypes. Interestingly, GP, M6, and M8 are resilient for PNUE whatever the N supply.

### Deciphering Groups of Barley Genotype Displaying Similar GxN Responses to N Supply

**FIGURE 2:**
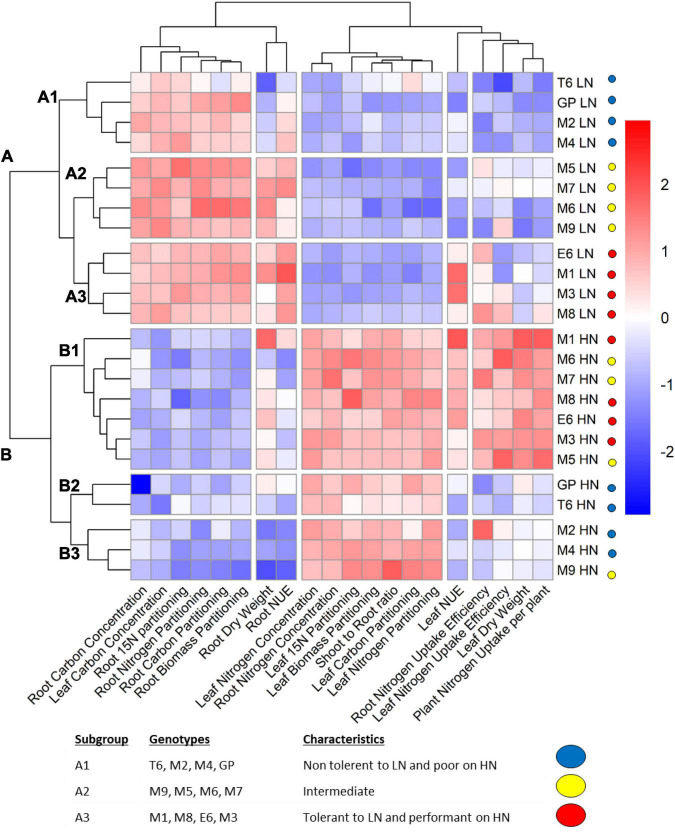
Hierarchical clustering analysis (HCA) showing groups of genotypes sharing similar physiological traits. Plants were grown for 14 days under LN or HN then leaves and roots were harvested separately and frozen under liquid nitrogen. The physiological parameters were measured as indicated in the “Materials and Methods” section. The color scale is based on the value of the normalized mean for each trait. Normalization was made for the LN and HN conditions separately. The clustering under LN was chosen to determine three subgroups (A1, A2, and A3) labeled with the indicated colors. HCA was constructed with the R package.

To compare the traits between the barley genotypes and determine common patterns shared within the collection under LN and HN, a hierarchical clustering analysis (HCA) was applied to key physiological variables. This allowed us to identify the traits that displayed the most conserved trends and those that showed the highest variation among the genotypes. Genotypes presenting similar profiles could then be clustered (Figure 2).

Clustering clearly separated the two nutritional conditions (clusters A and B) indicating that nitrate supply is the main factor affecting barley physiological traits. Several traits displayed opposite trends in LN and HN for the whole barley collection. This clustering shows that most traits related to the roots reached higher values under LN than under HN, and the opposite is observed for the shoots. Interestingly, the traits that displayed genetic variation among the collection and reflected different responses to nitrate supply of the barley genotypes are essentially related to N uptake (leaf and root nitrogen uptake efficiency and total nitrogen uptake), leaf and root DW, and nitrogen use efficiency (leaf and root NUE) (Figure 2).

Under low N conditions (cluster A), genotypes are distributed into three groups. The A3 group is clearly the most efficient because it displays the best leaf NUE amongst the entire collection. The A3 genotypes have a bigger root system and higher root N uptake efficiency than the others. The A1 group is the less efficient based on leaf NUE which coincided with lower total N uptake per plant, lower leaf dry weight as well as lower leaf and root N uptake efficiencies. The A2 group displays intermediate characteristics with reduced capacity to use nitrogen resources to produce biomass. Indeed, what clearly distinguishes A2 from A3 is the leaf NUE.

Data from A group (plants under LN) highlight the different strategies of barley genotypes to deal with limiting nitrate as previously suspected from ANOVA (Figure 2 and Supplementary Figure 2). In the most performant genotypes (A3 group), the highest leaf NUE is associated with the highest root nitrogen uptake efficiency, root biomass, and root NUE. Taken together, the data from barley grown under low nitrate show that performance in N use is linked to high root biomass and high N uptake.

Under the HN condition (cluster B), three groups, B1, B2, and B3, display different behaviors. The B1 genotypes are characterized by high leaf and root N uptake, high leaf NUE, and higher leaf and root DW. These B1 genotypes are more performant than the others for nitrogen uptake, translocation, and assimilation; they efficiently use their N resources to produce biomass. The B2 and B3 genotypes are less performant. Indeed, in contrast with B1 genotypes, they exhibit lower N uptake, lower leaf NUE, and lower root dry weight. It is then interesting to focus on what distinguishes B2 from B3. In the B2 group, root biomass is more important than in the B3 group. However, in B2, N uptake in the root is lower than in B3, and as a consequence, there is a lack of N uptake in the shoots that display *per se* low leaf NUE. Then, B3 seems more performant than B2 since, with less root biomass, it can take up nitrogen more efficiently in both root and shoot. Taken together, data from the B group (plants under HN) highlight the different strategies developed by plants to use nitrate when it is not limiting. Performance for N utilization in the shoot is linked to larger roots and higher plant N uptake capacity. Some genotypes (M2, M4, M9) are able to fine-tune their leaf NUE with reduced root biomass.

Interestingly, genotypes with poor performance under LN (sub-cluster A1) also displayed poor performance under HN (B2/B3), and genotypes with high performances under LN (sub-cluster A3) also kept high performances under HN (B1). With the exception of GP and M9, all the other intermediate genotypes from sub-cluster A2 performed relatively better under HN indicating that these genotypes are less tolerant to low nitrate availability than the others. The A1 sub-cluster contains the T6, GP, M2, and M4 genotypes. Interestingly, M2 and M4 belong to B3 and GP and T6 to B2, indicating that they are poorly performant under both LN and HN.

We were able to identify four genotypes M1, M8, E6, and M3 that displayed good performance in both LN and HN. Genotypes that perform poorly under both LN and HN are T6, M2, and M4 due to their reduced root biomass and low N uptake.

Taken together, our data indicate that an increase in the root nitrogen sink strength and of global C content are the most conserved responses to nitrogen limitation among the studied genotypes. The most heterogeneous responses are related to N uptake efficiency and NUE, which highlight different metabolic adaptation strategies to N limitation. Dissecting the molecular mechanisms building such a diversity deserves further attention for a better comprehension of the genetic diversity of plant strategies for adaptation to nitrate limitation.

### Diversity of Amino Acid Concentrations in the Barley Collection Grown Under Low or Ample N Supply

Nitrogen metabolism is strictly related to amino acid composition, which can play diverse roles in plant physiology and tolerance to stress (Rai, 2002; Zeier, 2013; Galili et al., 2016). Thus, to better characterize the nitrogen metabolism in the barley collection, amino acid concentrations were determined in leaves and roots under LN and HN using HPLC.

**FIGURE 3:**
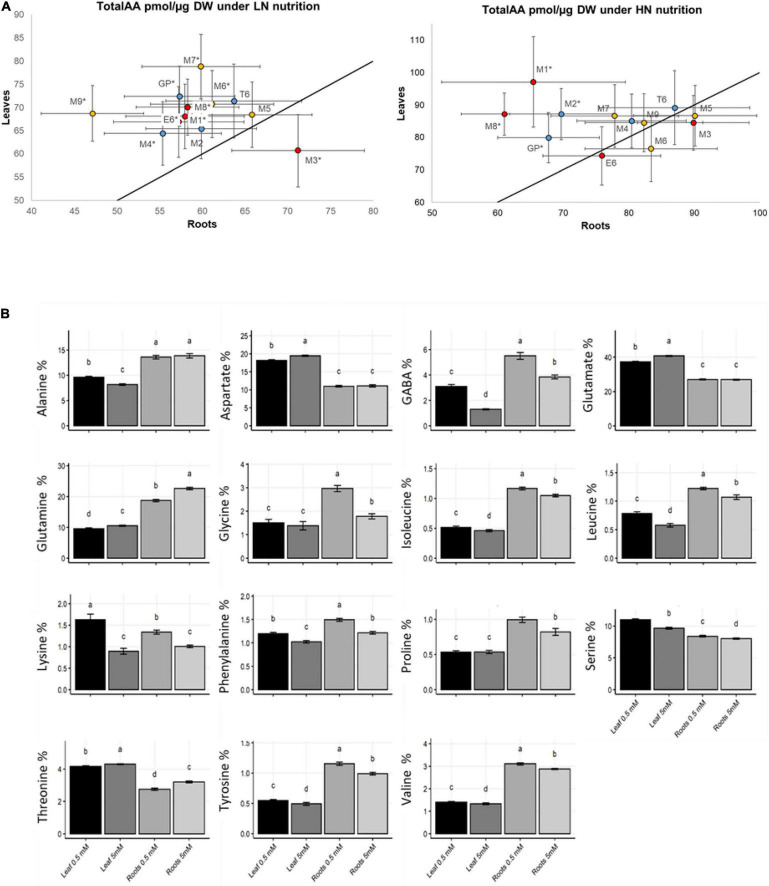
Amino acid distribution in barley leaves and roots under LN and HN. Plants were grown for 14 days under LN or HN then leaves and roots were harvested separately and frozen under liquid nitrogen. Following freezing in liquid nitrogen, AA were quantified by HPLC as indicated in the “Material and Methods.” **(A)** Comparison of total amino acids levels in leaves were plotted against levels in roots of barley genotypes under LN and HN as indicated. The strait line represents the Y = X curve. Bars represent SE. Stars indicate a significant difference between LN and HN for each barley genotype (Student’s test, 13 ≤ *n* ≤ 16, *p* < 0.05). Colors of the dots correspond to the classes defined in Figure 1. **(B)** Individual amino acid % in the barley collection under LN or HN. Four independent experiments were performed. Stars indicate significant difference between LN and HN (Student’s test, 13 ≤ *n* ≤ 16, *p* < 0.05). Bars represent SE.

As expected, total free amino acid concentration was significantly higher under HN than under LN in both shoot and root (Supplementary Figure 3). To know how amino acid distribution between aerial parts and roots is controlled in response to N supply, we compared total amino acid contents in leaves and roots for each genotype. All the genotypes accumulated higher amounts of amino acids in shoot than root under LN except M3 (Figure 3A). The contrast between root and shoot was less important under ample N supply and only four genotypes (GP, M1, M2, M8) contained significantly higher amounts of amino acids under HN.

Since amino acids have different roles in plant metabolism (Häusler et al., 2014), we investigated the influence of N supply on the concentration of individual amino acids. The relative proportion of each amino acid was calculated as % of total amino acids. Globally, the percentages of individual amino acids depended on the organ and the N nutrition (Figure 3B). For instance, in both shoot and root, accumulations of GABA branched-chain amino acids (BCAA: isoleucine, leucine, valine), phenylalanine, serine, tyrosine, and lysine under N limitation was paralleled with a decrease of the percentage of glutamine and threonine. Aspartate and glutamate percentage was also decreased under LN but only in leaves.

**FIGURE 4:**
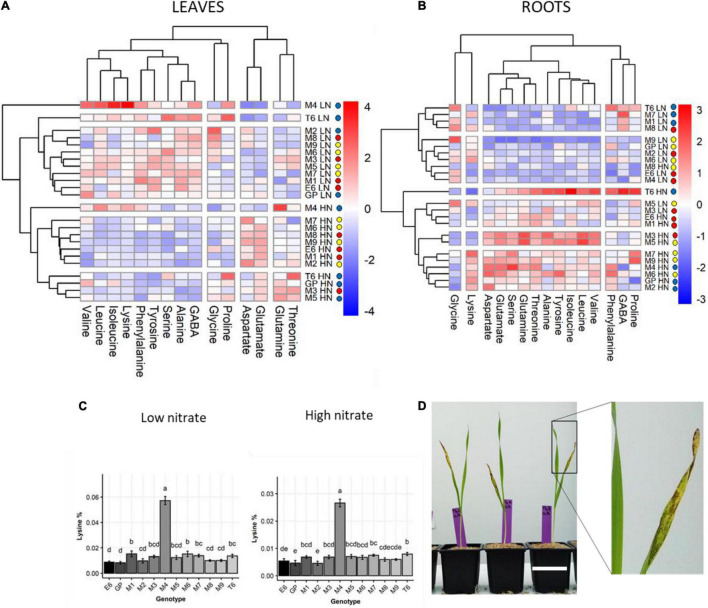
Genetic diversity of amino acid composition in barley leaves and roots under LN and HN. Plants were grown for 14 days under LN or HN then leaves and roots were harvested separately and frozen under liquid nitrogen. The following freezing in liquid nitrogen AA was quantified by HPLC as indicated in the Material and methods. **(A,B)** Hierarchical clustering analysis of the amino acid % under LN or HN in leaves and roots, respectively, showing genotypes sharing similar amino acid profiles. The color scale is based on the value of the normalized mean for each trait. Normalization was made for the LN and HN conditions separately. The colored circles in front of each genotype label represent the above-mentioned group A, B, C in Figure 2. HCA was constructed with the R package. **(C)** Level of lysine in leaves of each genotype under HN or LN. The different letters indicate values significantly different at *P* < 0.05 as determined using R-ANOVA Newman–Keuls (SNK) comparisons. **(D)** Picture showing the senescing phenotype of M4 leaves under LN. An enlargement of the senescing leaf is shown on the right. White scale bar = 5 cm.

Hierarchical clustering based on the relative proportions of individual amino acids was performed independently for the shoot (Figure 4A) and root (Figure 4B). In leaves, clustering clearly separated LN and HN. In the root, there was no HN or LN-dependent clustering.

Interestingly, HCA facilitated the identification of two genotypes (M4 and T6) that did not cluster with any other genotype irrespective of nutrition or organs (Figure 4). The M4 genotype indeed accumulated five times more lysine in shoot than any other genotype, irrespective of N conditions (Figure 4C). Under low N, M4 also displayed higher proportions of branched-chain amino acids (isoleucine, leucine, and valine) and proline in shoots compared to all the other genotypes (Figure 4A and Supplementary Figure 4). Under high N, in addition to lysine, proportions of glutamine, isoleucine, and leucine were also higher in the M4 shoot compared to other genotypes (Figure 4A and Supplementary Figure 5). The percentage of glutamate and aspartate in the M4 shoot were among the lowest irrespective of N nutrition. Interestingly, the M4 genotype displayed an early senescing phenotype on leaves 12 days after sowing under LN (Figure 4D) that may explain the special amino acid profile of this barley genotype.

The T6 genotype was also quite different from others. It exhibited low glutamate and aspartate percentage in shoot under LN and higher isoleucine, phenylalanine, proline, and leucine percentages (Figure 4A and Supplementary Figure 3). In the root, T6 is characterized by a higher percentage for most of the minor amino acids except lysine and aspartate under HN (Figure 4B and Supplementary Figure 4). Under LN, the T6 root did not distinguish itself from other genotypes. The most prominent amino acid feature of T6 is a higher percentage of serine and proline.

Clustering of the barley genotypes according to their amino acid profiles in roots or leaves (Figure 4) was different from clustering based on physiological traits (Figure 2). This suggests complex relationships between N assimilation and amino acid homeostasis.

### Transcriptional Changes in Limiting N Relative to Ample N Conditions

**FIGURE 5:**
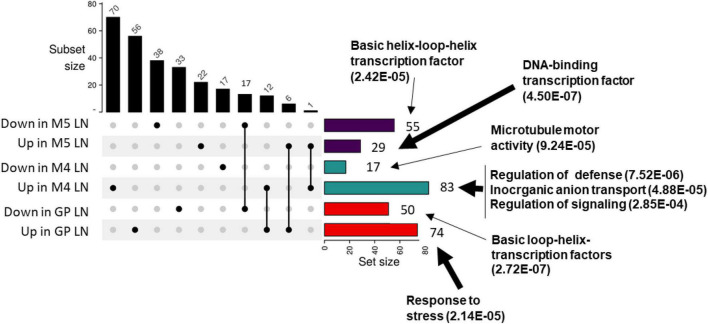
Differentially regulated genes under LN and HN in GP, M4, and M5. Plants were grown for 14 days under LN or HN then leaves were frozen under liquid nitrogen. RNA was extracted from leaves and RNAseq was performed as indicated in “Materials and Methods” section. Upset plot for overlapping up and down differentially expressed genes in M4, M5, and GP barley genotypes under LN or HN. The number of genes in each category is indicated on top of each bar. Functional categories overrepresented in the set of genes are indicated next to the arrows (geneontology.org). Thick arrows indicate up-regulated genes, thin arrows indicate down-regulated genes.

To further characterize the molecular processes taking place in barley in response to nitrate limitation, an RNAseq transcriptomic approach was undertaken on leaves of three barley genotypes displaying different physiological responses to N supply: GP, M4, and M5. This approach is aimed at identifying genes that are related to barley adaptation to nitrate limitation. The rationale behind the choice of these three genotypes was the following. First, the M4 genotype displays very poor adaptation to low N with early senescing leaves under LN while the M5 genotype had intermediate N adaptation traits under LN with high leaf and root N uptake efficiencies and leaf and root biomass under LN as shown in HCA (Figures 2, 4). The GP genotype was included since it is one of the most used genotypes in barley genomics studies. In addition, the poor response of GP PNUE to N availability is a shared feature with M5 (Figure 1). Significant GO overrepresented functions encoded by genes differentially expressed in the three genotypes were found to be related to stress responses, defense, signaling, and cytoskeleton remodeling (Figure 5). The differential regulation of defense-related genes prompted us to test the impact of N on barley tolerance to *Pyrenophora teres* Drechsler (anamorph *Drechslera teres*) one of the major pathogens affecting barley especially in Morocco (Jebbouj and El Yousfi, 2009; Backes et al., 2021a). Disease severity was higher under HN compared to LN in M5 and GP (Supplementary Figure 6).

In order to determine genes differentially expressed in the three genotypes and that could be candidate markers for nitrate limitation, we looked for genes commonly regulated in the three genotypes in LN vs. HN. There were no common genes with similar expression profiles in the three genotypes. However, 12 genes were up-regulated both in GP and M4 (Supplementary Table 4). They encode functions related to senescence, stress response, and ionic transport. Interestingly, the nitrate transporter encoding gene annotated *HvNRT2.10* (HORVUHr098550 orthologous to *AtNRT2.7*) is up-regulated in GP and M4. Although not statistically significant, we could observe an up-regulation of this gene in M5 LN compared to HN with a *p*-value close to the level of significance (*p* = 0.06). These data suggest that *HvNRT2.10* is commonly up-regulated in the three genotypes further supporting this gene as a candidate involved in nitrate nutrition under N limitation. Six genes were found to be commonly down-regulated in GP and M5, three of them encode transcription factors, and the three others encode iron-containing proteins (Supplementary Table 5).

Together, these data indicate that functions related to stress, immunity, signaling, senescence, and ionic transport are affected by N limitation in barley.

### Genotypic Diversity of Barley Transcriptome Supports Amino-Acid Profiles

**FIGURE 6:**
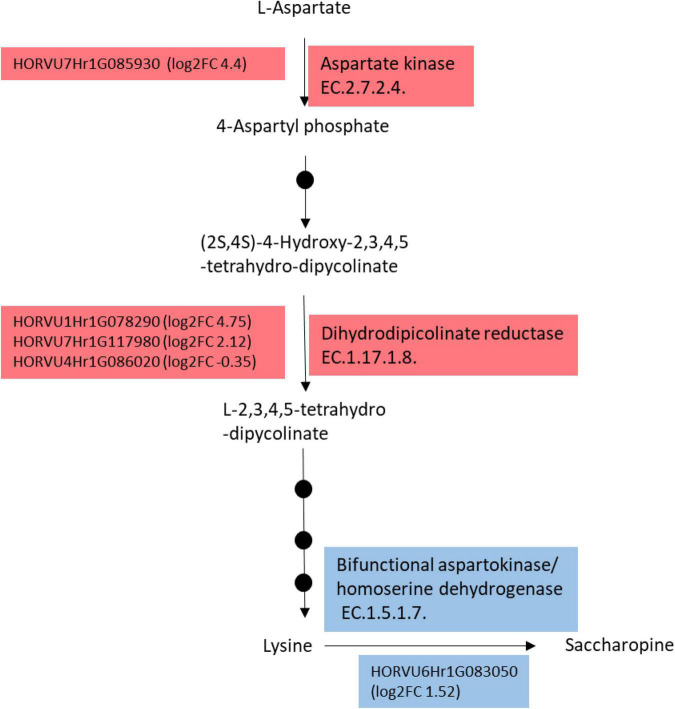
Simplified Lysine biosynthesis and catabolism pathways were found to be differentially expressed in M4 compared to GP and M5. Genes encoding enzymes of these pathways are indicated with their accession numbers in front of the corresponding enzyme. For each gene, the log2 of the fold change (log2FC) corresponds to the expression in M4 compared to the mean of the gene expression level in M5 and GP. Red and blue boxes correspond to biosynthesis and catabolism of lysine, respectively. Black dots represent intermediate enzymatic steps that were omitted for simplification.

Since we found that lysine was highly accumulated in M4 leaves compared to the other genotypes, we investigated genes involved in the lysine metabolic pathway in the transcriptome of M4 compared to the two other genotypes M5 and GP. For this purpose, the transcriptomic profile of M4 was compared to the average of the transcript levels of each gene in GP and M5 (hereafter referred to as “GP+M5”) under HN because of the variance of the transcriptome under HN was lower than under LN (data not shown). Lysine is synthesized through a branch of the Asp family pathway. The first reactions leading to lysine biosynthesis (Jander and Joshi, 2009) are catalyzed by aspartate kinase, dihydrodipicolinate synthase, and reductase. At least eight genes putatively involved in lysine biosynthesis, degradation, and transport were differentially expressed in M4 compared to M5+GP. Two genes encoding putative dihydrodipicolinate reductase (*HORVU1Hr1G078290* and *HORVU7Hr1G117980*) are up-regulated in M4 and a third one putatively encoding the same enzyme (*HORVU4Hr1G086020*) was down-regulated (Figure 6). A gene encoding a putative aspartate kinase (*HORVU7Hr1G085930*) and three genes encoding putative lysine histidine transporter 1 (*HORVU2Hr1G123160*, *HORVU7Hr1G074640*, and *HORVU7Hr1G074660*) were found to be up-regulated in M4 (see RNAseq data). In addition, a gene encoding the bifunctional Lys-ketoglutarate reductase/saccharopine dehydrogenase was found to be upregulated in M4 compared to GP+M5 (*HORVU61G083050*). In addition to lysine accumulation, M4 leaves accumulate higher levels of BCAA (leucine, isoleucine, and valine, Figure 4 and Supplementary Figures 4, 5). Consistently, genes encoding three key steps involved in BCAA biosynthesis were found to be differentially expressed in M4 compared to GP+M5 as follows (Binder et al., 2007; Binder, 2010). The branched-chain amino acid transaminase encoding genes *HORVU2Hr1G096380*, *HORVU3Hr1G032400* are upregulated in M4 by a log2 fold change (log2 FC) of 1 and 1.4, respectively. The threonine aldolase encoding genes *HORVU2Hr1G097910* and *HORVU4Hr1G085690* are down-regulated in M4 by a log2 FC of -.53 and -.6, while *HORVU1Hr1G046630* is up-regulated by a log2 FC of 1.26. The isopropylmalate dehydrogenase encoding genes *HORVU2Hr1G124400*, *HORVU3Hr1G059060*, *HORVU3Hr1G000570* are up-regulated in M4 by a log2 FC of 0.35, 3.35, and 0.54, respectively while *HORVU7Hr1G066450* is down-regulated in M4 by a log2 FC of -6.58.

Thus, transcriptomic data are consistent with the M4 phenotype and its amino acid composition profile.

## Discussion

Owing to the large genetic diversity of barley and its resilience under harsh environments, this crop is of great value for agroecological transition under global change and the need for reduction of nitrogen fertilizers inputs. Barley culture in north Africa is mainly performed under rainfall conditions and nitrogen input is a limiting factor to the same extent as water availability (Ryan et al., 2008; Ryan and Sommer, 2012). Indeed, North African soils are calcareous with low organic matter content thus requiring N fertilization (Ryan and Sommer, 2012). However, for economic and environmental reasons, it is crucial to improve the management of N fertilization. Therefore, it is crucial to characterize the Mediterranean varieties with respect to their adaptation to different nitrogen supplies. We worked on a panel of north African barley genotypes thus, adapting to the Mediterranean climate and environment.

Our goal was to focus on the response of these genotypes to low N and to decipher the diversity of their physiological and molecular responses to N supply at early stages of development. Here, we have considered that the most performant genotypes are those displaying higher leaf NUE under LN.

Traits affected by N supply at the level of the whole barley collection are an increase in C content in the whole plant and increased root biomass. Partitioning of root C, N, and biomass was increased in roots compared to leaves under LN. These are well-known responses of plants to LN and the major role of root in this response is well-known (Lea and Miflin, 2018; Sun X. et al., 2020). Interestingly, leaf carbon concentration was positively correlated with several root traits (root NUE, root ^15^N partitioning, root carbon partitioning, and root biomass partitioning) (Supplementary Figure 1). Similarly, positive correlations were observed between leaf and root N uptake efficiencies indicating coordination of both processes. In addition, leaf biomass was positively correlated with plant nitrogen uptake and leaf NUE indicating an important role of N uptake and utilization in building the aerial biomass of barley plants (Supplementary Figure 1). Root allocation of C and N is generally observed as a general response to N deficiency (Zhang et al., 2007; Kobe et al., 2010; Krapp et al., 2011). Root DW was higher under LN compared to HN, which globally resulted in a decrease of the shoot/root ratio (SR) under LN, in good accordance with previous reports (Van Der Werf and Nagel, 1996; Lea and Azevedo, 2007). In contrast, carbon concentration was higher under LN which is consistent with the fact that under N deficiency, plants usually accumulate sugars, starch, or fructan (Wang et al., 2000; Lemaître et al., 2008). Here we verified that NUE was higher under LN than under HN which is a shared feature with other plant species (Chardon et al., 2010; Masclaux-Daubresse and Chardon, 2011; Lammerts van Bueren and Struik, 2017). Higher NUE in LN-grown plants is explained by the fact that low N supply results in a tradeoff that favors the use of metabolic resources to support growth and survival. Conversely, when nitrogen is not limiting, a proportion can be stored under the form of nitrate in vacuoles and is not directly metabolized. These data show that nitrate nutrition significantly affects all traits except root dry weight, in good accordance with the physiological responses to N limitation previously reported (Van Der Werf and Nagel, 1996; Lea and Azevedo, 2007). These data make sense since the capacity of larger root systems to better explore the soil allows a higher nitrate uptake and a more efficient translocation of inorganic nitrogen to the shoot available for growth and biomass production (Gastal and Lemaire, 2002).

Other traits display different variations depending on the barley genotype in response to nitrogen supply highlighting a GxN interaction: root development and nitrogen uptake processes. For instance, root dry weight increased under LN for some genotypes while it was lower under LN for other genotypes. Similar trends were observed for maize where LN affected shoot biomass negatively but had different impacts on root biomass indicating that root plasticity allows a reliable marker of adaptation to LN (Chun et al., 2005). Root growth under LN is known to be mainly due to increased auxin levels in the root but this may be counteracted by the action of other hormones mainly abscisic acid, ethylene, and cytokinin (Sakakibara et al., 2006; Sun X. et al., 2020). Thus, different root developments in the barley genotypes in response to N limitation may reflect different hormonal regulatory mechanisms. The diversity of physiological responses allowed us to classify the barley genotypes into three categories; tolerant, moderately tolerant, and poorly tolerant to LN based on their leaf NUE. Interestingly the two genotypes GP and T6 originating from Europe and Tunisia, respectively, exhibited lower root biomass under LN and low leaf NUE. It remains to be determined whether this classification is also observed in the field.

The investigation of the impact of N supply on the transcriptome of three barley genotypes led to the identification of a low number of differentially expressed genes compared to other studies (Comadira et al., 2015; Quan et al., 2019). Nevertheless, significant GO overrepresented functions encoded by genes differentially expressed in the three genotypes were found to be related to stress responses and to signaling (Figure 5). Down-regulated genes in M4 under LN were related to microtubule-binding motor protein suggesting a down-regulation of cell vesicular trafficking and/or an arrest in cell development. Signaling, ionic transport, and metal enzymes are common over-represented functional categories in our study and in the aforementioned transcriptomic analyses. The differential expression of genes related to defense in the RNAseq is in agreement with the observed impact of N supply on barley susceptibility to one of the major pathogens affecting barley especially in Morocco (Jebbouj and El Yousfi, 2009; Backes et al., 2021a). It is known that N nutrition can affect plant tolerance to pathogens but positive and negative correlations have been observed depending on the plant-pathogen interaction considered (Fagard et al., 2014; Mur et al., 2017; Sun Y. et al., 2020). In the case of the barley-*P. teres* interaction, N enhances susceptibility.

The low number of differentially expressed genes found in the present study may be due to the long-lasting stressful conditions experienced by the plants from seed sowing to harvest under LN. Indeed, at the time of harvest, i.e., 14 days after sowing, most of the metabolic processes may have been adjusted and stabilized under LN and HN. Among the genes found to be commonly up-regulated, the putative nitrate transporter coding gene *HvNRT2.10* (HORVUHrG098550) orthologous to the Arabidopsis *AtNRT2.7* gene, is of particular interest since it was found in a QTL mapping study as involved in barley tolerance to low N (Karunarathne et al., 2020). This gene is closely related to *OsNRT2.4* (Guo et al., 2020) which encodes a dual affinity nitrate transporter and was found to be involved in rice N nutrition although no phenotype was found for the knockout mutant (Wei et al., 2018). Further investigation of the function *of HvNRT2.10* in barley nitrogen nutrition deserves attention.

Nitrogen limitation altered amino acid composition in leaves and roots. The overall soluble amino acid concentration decreased under LN. While roots appeared as C and N metabolic sinks under LN for most genotypes, partitioning of soluble amino acids in roots varied depending on the genotype. Most genotypes concentrated amino acids in leaves under LN while this partitioning was more diverse under HN suggesting variability for the role of amino acids in barley coping with N deficiency.

Interestingly, leaf amino acid composition was correlated with nitrogen supply. Indeed, we found that the profiles of amino acids under LN were strictly different from the profiles under HN. Thus, an important impact of nitrogen nutrition can be observed in the aerial part of the plant. Notwithstanding the nutritional effect, an important genetic diversity of relative amino acid composition was observed between barley genotypes.

Nitrate limitation resulted in elevated levels of the amino acids GABA, Tyr, Leu, Ileu, Val, Phe, Ser, Lys in roots and leaves of the barley plants. In addition to being vital components of proteins, these amino acids display additional properties, such as signaling, stress tolerance or provide precursors for other compounds. For instance, GABA is known to be involved in plant stress tolerance to biotic and abiotic stresses (Ramesh et al., 2017; Xu et al., 2021). More specifically, GABA was described as triggering a better N uptake under stress conditions, such as salt stress or N limitation (Chen et al., 2020; Khanna et al., 2021). BCAA (Leu, Ileu, Val) are known to accumulate in response to abiotic stresses presumably to serve as a substrate for biosynthesis of stress proteins (Joshi et al., 2010). They are also known to serve as substrates in the biosynthesis of lipids and glucosinolates (Binder et al., 2007). Tyrosine accumulation in barley genotypes in response to LN may be linked to the role of this amino acid as a precursor for several products that could be involved in response to low N, such a tocopherol providing an antioxidant effect, or electron carrier or defense compounds (Schenck and Maeda, 2018; Xu et al., 2020). Phenylalanine is the precursor of phenylpropanoids known to be involved in tolerance to biotic stresses (Lynch and Dudareva, 2020). Accumulation of stress-related amino acids is consistent with the RNAseq data showing enhanced stress response signatures in LN barley leaves compared to HN. Interestingly, serine accumulation is mainly produced via increased photorespiration rate which is known to be able to provide ammonia under nitrogen deficiency (Shi and Bloom, 2021).

The investigation of amino acid content in this barley collection revealed that M4 stood out with a high lysine content. Interestingly, lysine was part of the amino acids accumulating in all genotypes under LN but reached five times higher levels in M4. In another study investigating amino acid content in four barley varieties, the authors found diversity in the lysine content of grains (Jood and Singh, 2001). In maize, the opaque mutant was identified as accumulating 69% more lysine in its endosperm compared to the parental line (Mertz et al., 1964; Wang et al., 2019). Interestingly, lysine accumulation in the endosperm is related to reduced levels of endosperm proteins like alpha-zein in maize (Wang et al., 2019) and hordein in barley (Schmidt et al., 2015). The level of lysine in M4 grains was not higher than that of M5 or GP (data not shown).

Lysine biosynthetic and catabolic pathways were extensively studied in plants because this is amino acid cannot be synthesized by human or monogastric bodies and it is present in low amounts in cereals (Galili, 2002; Galili et al., 2016). The key enzymes required for its biosynthesis in plants have been identified: dihydrodipicolinate synthase (DHPS) and the catabolic enzyme bifunctional Lys-ketoglutarate reductase/saccharopine dehydrogenase (LKR/SDH) (Galili et al., 2016). To increase the level of lysine, several approaches using DHPS overexpression or down-regulation of LKR/SDH or both were successful (Galili et al., 2016). Mutant forms of DHPS from *Nicotiana sylvestris* protoplasts resulted in lysine accumulation due to the loss of DHPS negative feedback regulation by lysine (Negrutiu et al., 1984). Strikingly, M4 transcript levels of genes encoding the two limiting steps in lysine biosynthesis and turnover, DHPS and LKR/SDH, are up-regulated compared to M5 and GP, suggesting that these genes are responsible for high lysine levels in M4. Thus, the accumulation of lysine in M4 might be due to altered negative feedback regulation of the DHPS enzyme. In addition, BCAA (leucine, isoleucine, and valine) accumulate to higher levels in M4 in agreement with the upregulation in the genotype M4 of genes encoding key enzymes involved in the biosynthesis of BCAA, the branched-chain amino acid transaminase, and the isopropylmalate dehydrogenase (Binder et al., 2007; Binder, 2010). One gene encoding a putative isopropylmalate dehydrogenase (*HORVU3Hr1G069300*) is down-regulated in M4 suggesting a fine-tuned regulation of this biosynthetic pathway depending on the isoforms. Interestingly, two genes encoding the threonine aldolase are downregulated. This is consistent with the reports indicating that this enzyme competes for threonine, the first amino acid in the BCAA biosynthesis pathway (Joshi et al., 2006).

Our work provides key physiological markers of North African barley adaptation to low N availability in the early developmental stages, in particular the *HvNRT2.10* gene. Candidate genes involved in key steps of lysine metabolism were identified with a potential link with immunity. Further investigation of the role of these genes in barley nitrogen metabolism and immunity would provide valuable data for sustainable barley production under harsh conditions.

## Data Availability Statement

RNA-Seq projects were deposited [Gene Expression Omnibus (Edgar et al., 2002)]: http://www.ncbi.nlm.nih.gov/geo/; accession no. GEO GSE188216. All steps of the experiment, from growth conditions to bioinformatic analyses, were detailed in CATdb (Gagnot et al., 2008): http://tools.ips2.universite-paris-saclay.fr/CATdb/project.html?project_id=493; project: NGS2020_19_ARIMNet according to the MINSEQE “minimum information about a high-throughput sequencing experiment”.

## Author Contributions

CM-D and AD: supervision of this work. FS, MB, and MR: plant growth, ^15^N labeling, and sampling. MB, MR, and SL: plant material preparation for all measurements. AM: ^15^N, N, and C quantification. MA and MB: amino acid determination. CP-L and JC: RNAseq. CJ and QE: pathogenicity tests. AD, BD, CM-D, FC, and MB: data computing and statistical analyses. AD, CM-D, and FC: project supervision and writing. All authors contributed to the article and approved the submitted version.

## Conflict of Interest

MA was employed by company TIMAC AGRO International SAS. The remaining authors declare that the research was conducted in the absence of any commercial or financial relationships that could be construed as a potential conflict of interest.

## Publisher’s Note

All claims expressed in this article are solely those of the authors and do not necessarily represent those of their affiliated organizations, or those of the publisher, the editors and the reviewers. Any product that may be evaluated in this article, or claim that may be made by its manufacturer, is not guaranteed or endorsed by the publisher.
