# Corrigendum: Genotypic Variation of Nitrogen Use Efficiency and Amino Acid Metabolism in Barley

**DOI:** 10.3389/fpls.2022.893540

**Published:** 2022-04-05

**Authors:** Bérengère Decouard, Marlène Bailly, Martine Rigault, Anne Marmagne, Mustapha Arkoun, Fabienne Soulay, José Caïus, Christine Paysant-Le Roux, Said Louahlia, Cédric Jacquard, Qassim Esmaeel, Fabien Chardon, Céline Masclaux-Daubresse, Alia Dellagi

**Affiliations:** ^1^Université Paris-Saclay, INRAE, AgroParisTech, Institut Jean-Pierre Bourgin (IJPB), Versailles, France; ^2^Agro Innovation International - Laboratoire Nutrition Végétale, TIMAC AGRO International SAS, Saint Malo, France; ^3^Université Paris-Saclay, CNRS, INRAE, University of Évry Val d′Essonne, Institute of Plant Sciences Paris-Saclay (IPS2), Orsay, France; ^4^Université de Paris, CNRS, INRAE, Institute of Plant Sciences Paris-Saclay (IPS2), Orsay, France; ^5^Natural Resources and Environment Lab, Faculté Polydiscipliniare de Taza, Université Sidi Mohamed Ben Abdellah, Taza, Morocco; ^6^Université de Reims Champagne Ardenne, RIBP EA 4707 USC INRAE 1488, SFR Condorcet FR CNRS 3417, Reims, France

**Keywords:** NUE (nitrogen use efficiency), crop/stress physiology, barley, natural variability, lysine (amino acids)

In the original article, there was an error in the **Funding statement**. The correct number for BEST is “BEST ANR-15-ARM2-0007”.

Here is the correct funding text:

“This work was supported by ARIMNET 2 project entitled “Exploring genotypic diversity to optimize barley grain and straw quality under marginal/stressful growth conditions,” BEST ANR-15-ARM2-0007. IJPB benefits from the support of the LABEX Saclay Plant Sciences-SPS (ANR-10-LABX-0040-SPS).”

The authors apologize for this error and state that this does not change the scientific conclusions of the article in any way. The original article has been updated.

## Publisher's Note

All claims expressed in this article are solely those of the authors and do not necessarily represent those of their affiliated organizations, or those of the publisher, the editors and the reviewers. Any product that may be evaluated in this article, or claim that may be made by its manufacturer, is not guaranteed or endorsed by the publisher.

