# Supplementary material for: Genotypic Variation of Nitrogen Use Efficiency and Amino Acid Metabolism in Barley
Source: Front Plant Sci. 2022 Feb 4;12:807798. doi: 10.3389/fpls.2021.807798 (PMC8854266; doi:10.3389/fpls.2021.807798)
Supplement: Supplementary file 1 [file Data_Sheet_1.zip › New folder/Supplementary Figure 1.PPTX]

## Slide 1
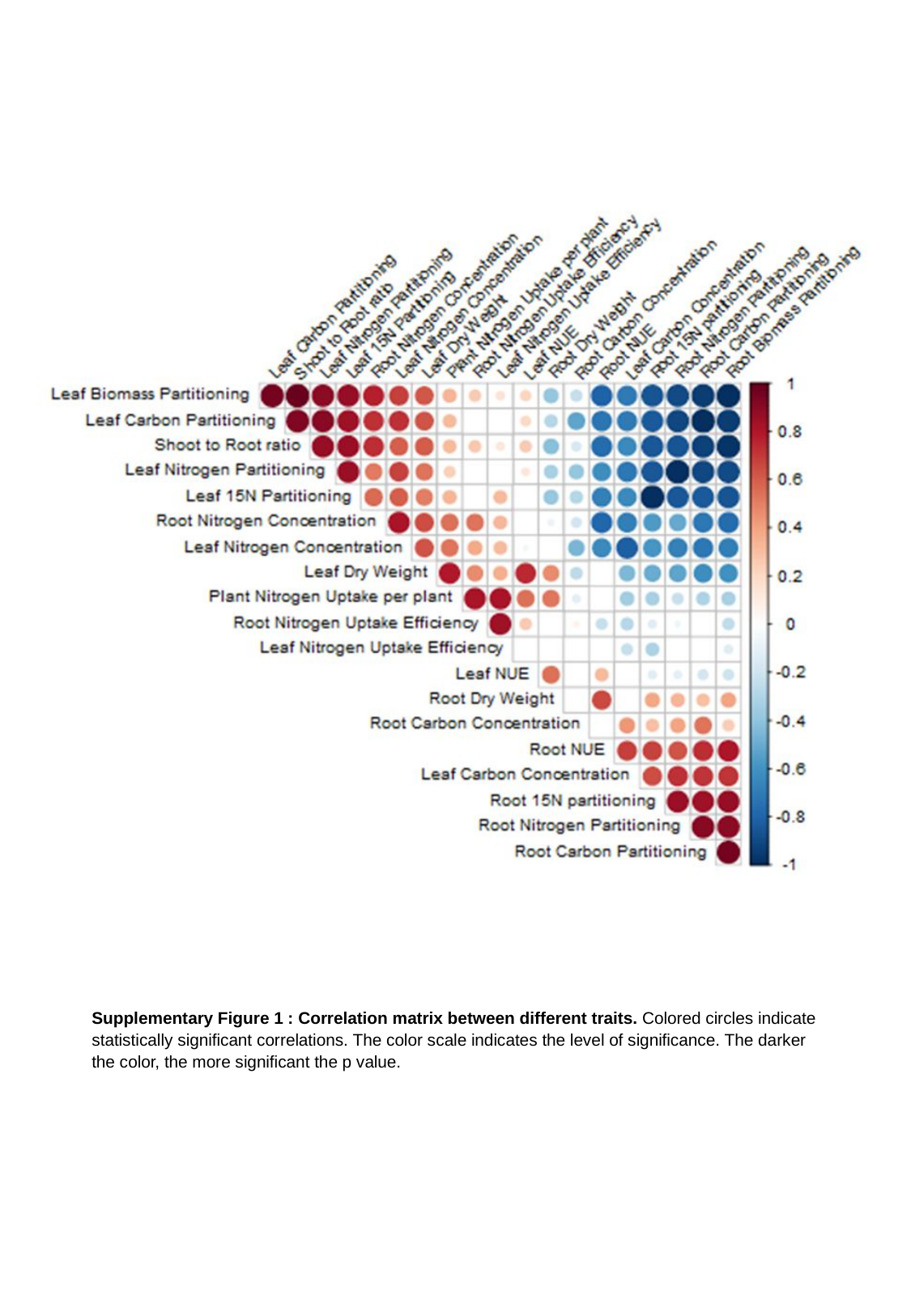

Supplementary Figure 1 : Correlation matrix between different traits. Colored circles indicate statistically significant correlations. The color scale indicates the level of significance. The darker the color, the more significant the p value.
