# Supplementary material for: Genotypic Variation of Nitrogen Use Efficiency and Amino Acid Metabolism in Barley
Source: Front Plant Sci. 2022 Feb 4;12:807798. doi: 10.3389/fpls.2021.807798 (PMC8854266; doi:10.3389/fpls.2021.807798)
Supplement: Supplementary file 1 [file Data_Sheet_1.zip › New folder/Supplementary Figure 2.PPTX]

## Slide 1
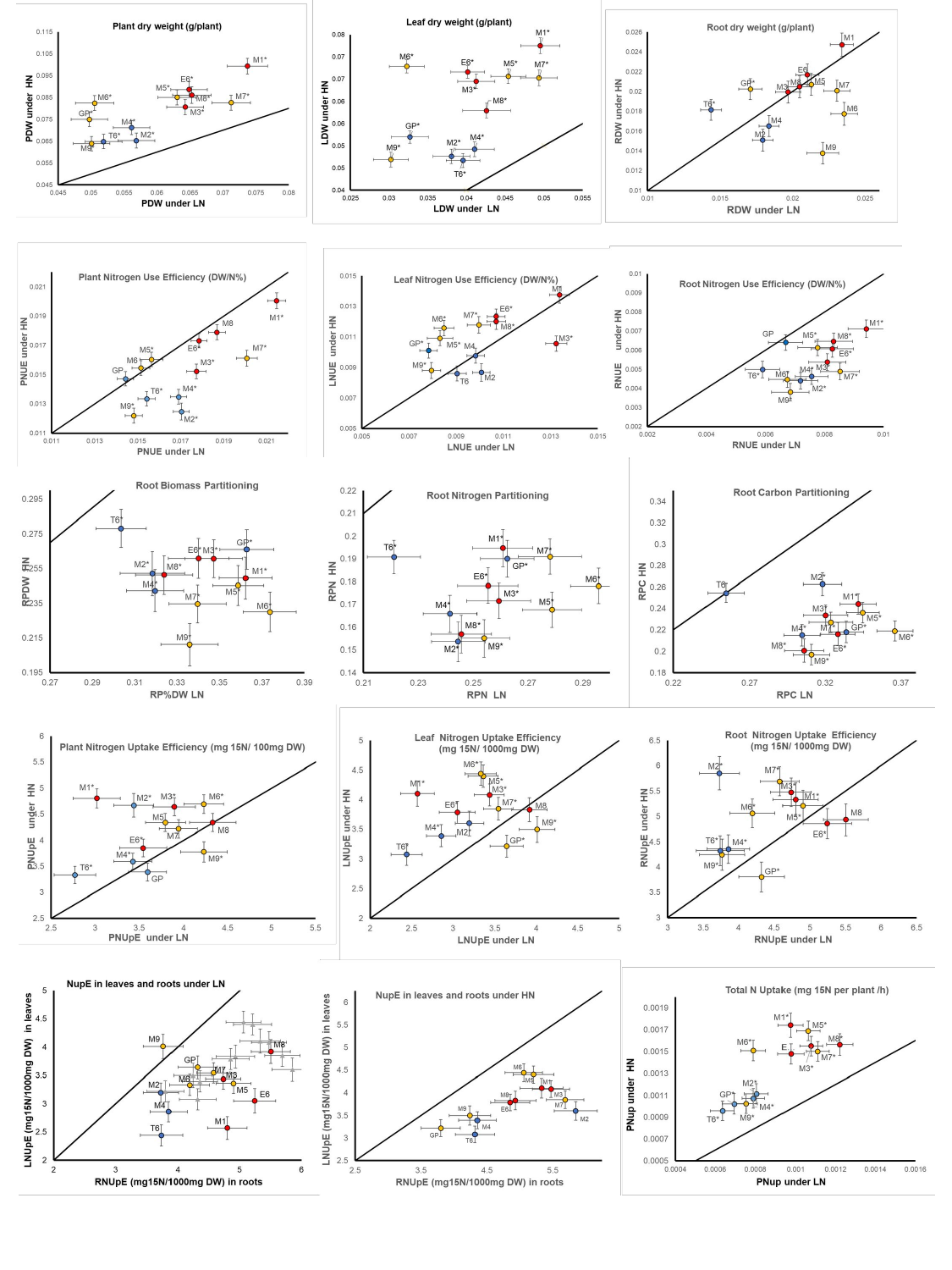

## Slide 2
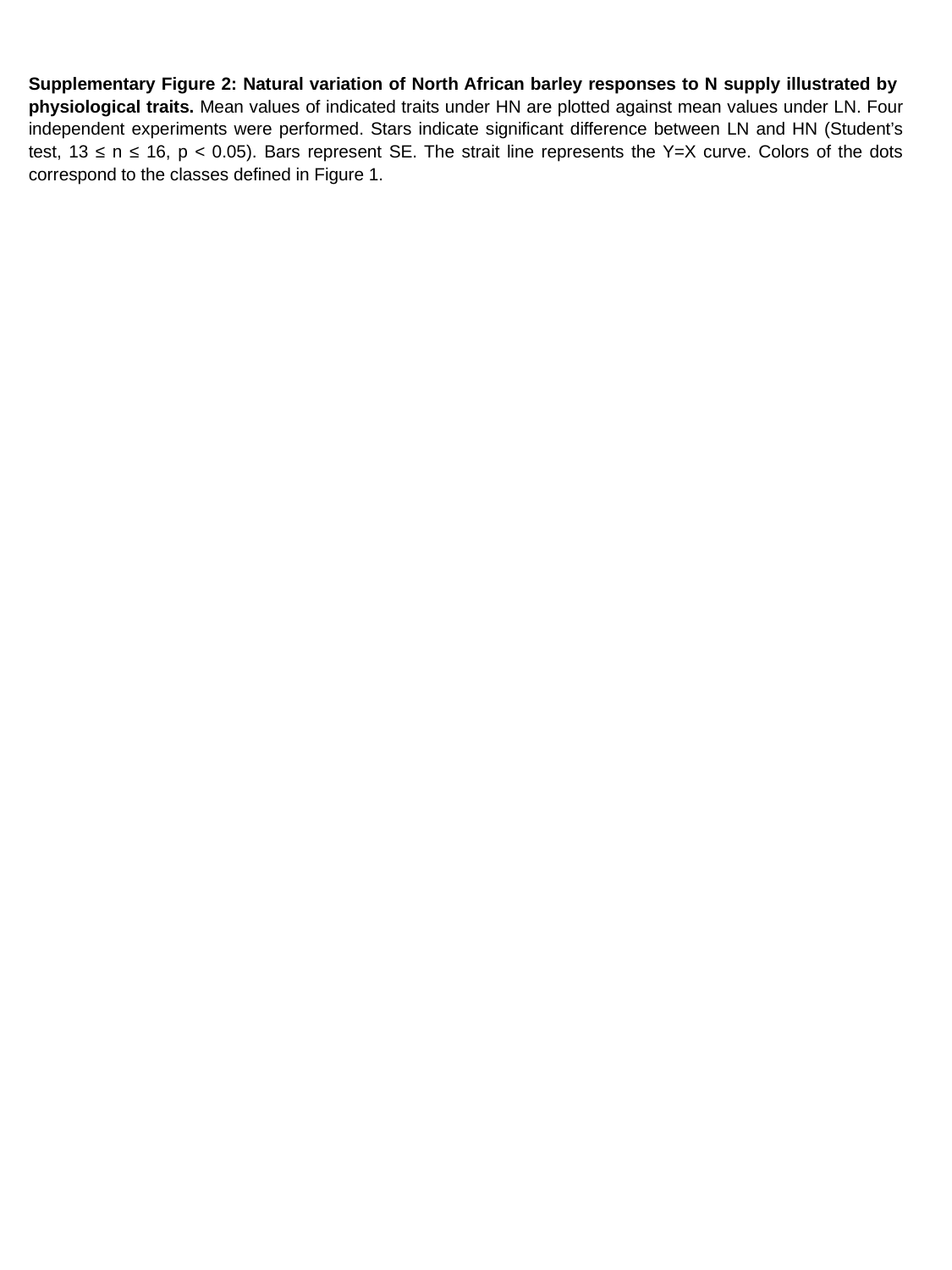

Supplementary Figure 2: Natural variation of North African barley responses to N supply illustrated by physiological traits. Mean values of indicated traits under HN are plotted against mean values under LN. Four independent experiments were performed. Stars indicate significant difference between LN and HN (Student’s test, 13 ≤ n ≤ 16, p < 0.05). Bars represent SE. The strait line represents the Y=X curve. Colors of the dots correspond to the classes defined in Figure 1.
