# Supplementary material for: Genotypic Variation of Nitrogen Use Efficiency and Amino Acid Metabolism in Barley
Source: Front Plant Sci. 2022 Feb 4;12:807798. doi: 10.3389/fpls.2021.807798 (PMC8854266; doi:10.3389/fpls.2021.807798)
Supplement: Supplementary file 1 [file Data_Sheet_1.zip › New folder/Supplementary Figure 5 Continued.PPTX]

## Slide 1
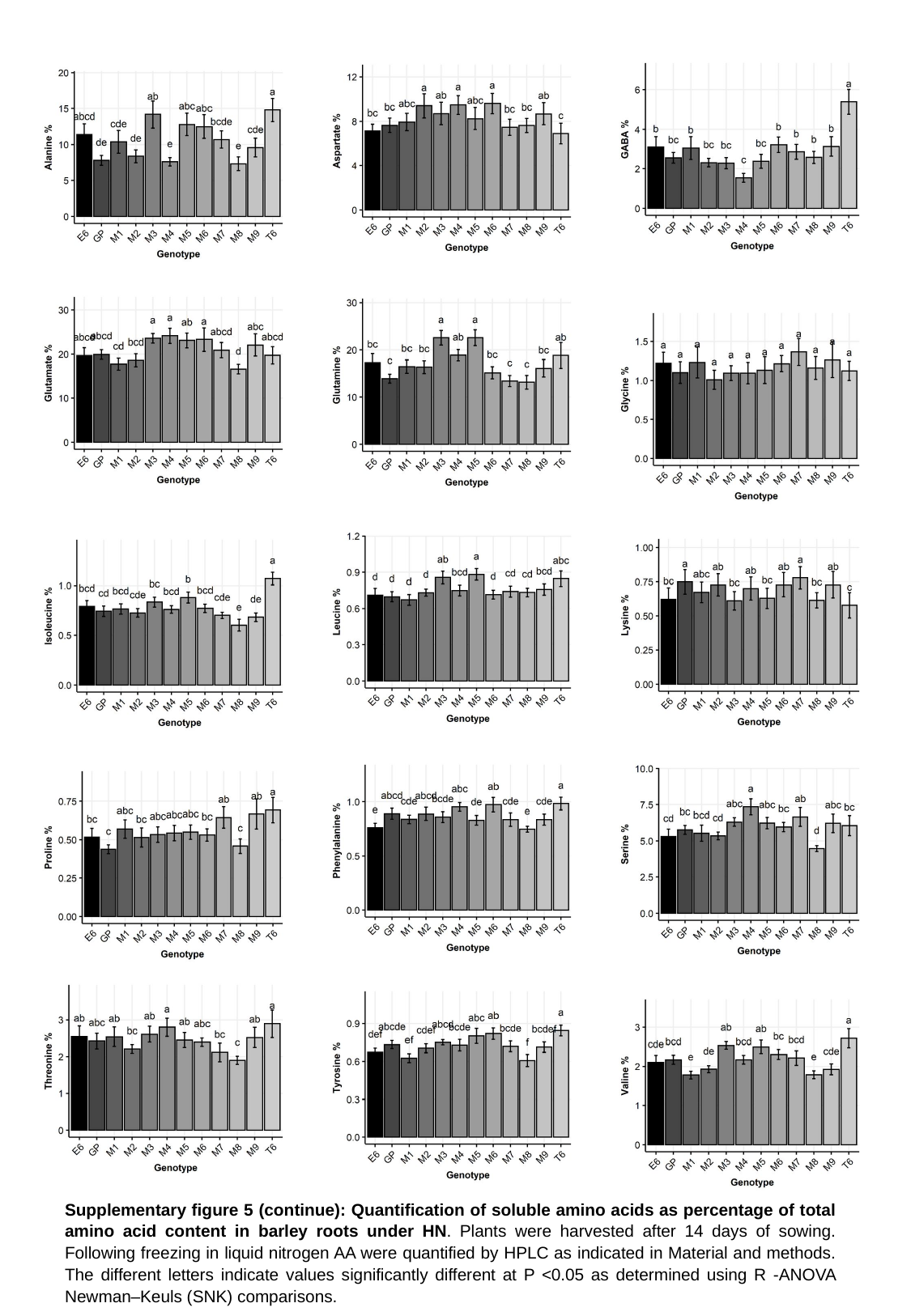

Supplementary figure 5 (continue): Quantification of soluble amino acids as percentage of total amino acid content in barley roots under HN. Plants were harvested after 14 days of sowing. Following freezing in liquid nitrogen AA were quantified by HPLC as indicated in Material and methods. The different letters indicate values significantly different at P <0.05 as determined using R -ANOVA Newman–Keuls (SNK) comparisons.
