# Supplementary material for: Genotypic Variation of Nitrogen Use Efficiency and Amino Acid Metabolism in Barley
Source: Front Plant Sci. 2022 Feb 4;12:807798. doi: 10.3389/fpls.2021.807798 (PMC8854266; doi:10.3389/fpls.2021.807798)
Supplement: Supplementary file 1 [file Data_Sheet_1.zip › New folder/Supplementary Figure 6.PPTX]

## Slide 1
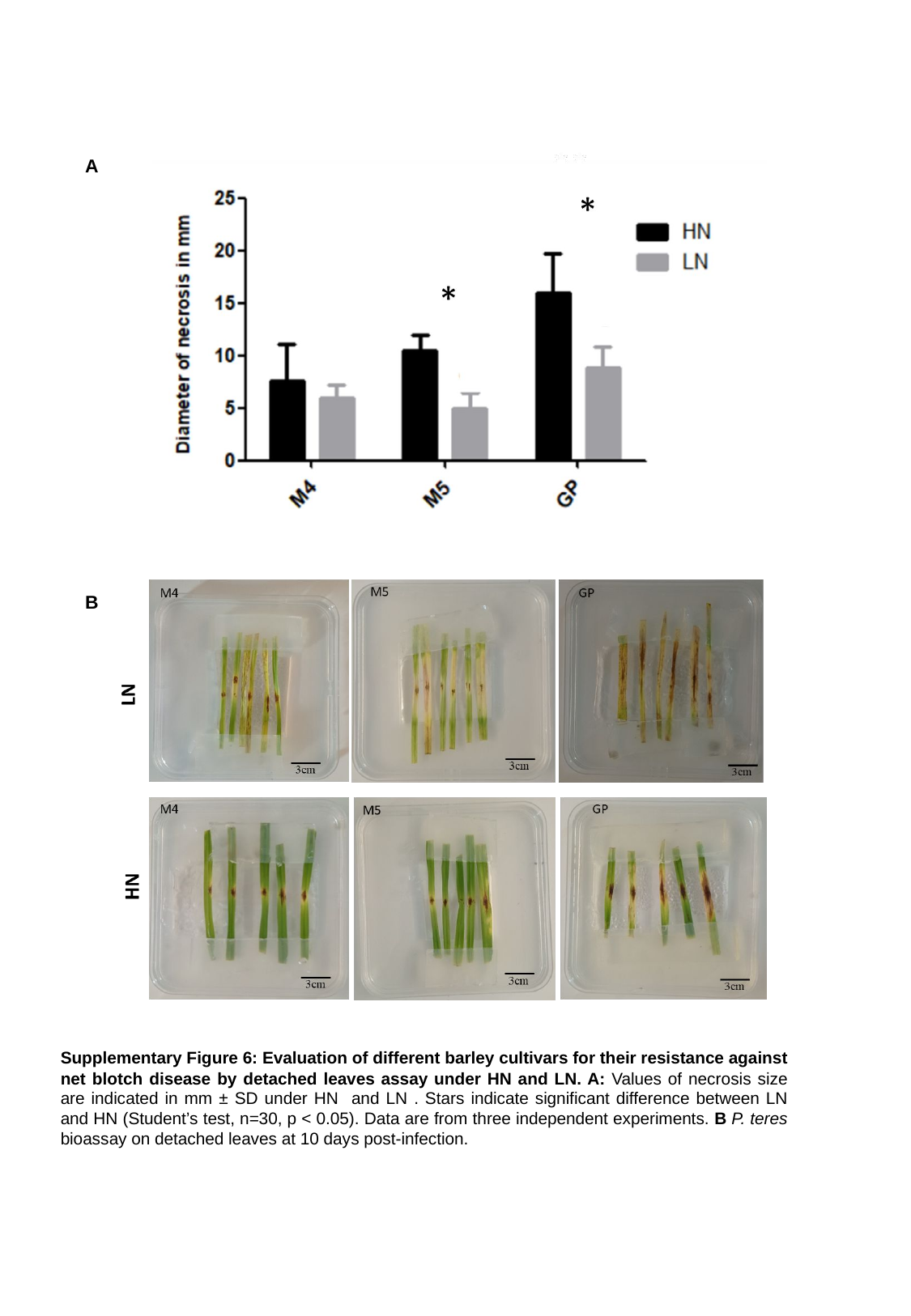

A
B
Supplementary Figure 6: Evaluation of different barley cultivars for their resistance against net blotch disease by detached leaves assay under HN and LN. A: Values of necrosis size are indicated in mm ± SD under HN and LN . Stars indicate significant difference between LN and HN (Student’s test, n=30, p < 0.05). Data are from three independent experiments. B P. teres bioassay on detached leaves at 10 days post-infection.
