# Supplementary material for: Genotypic Variation of Nitrogen Use Efficiency and Amino Acid Metabolism in Barley
Source: Front Plant Sci. 2022 Feb 4;12:807798. doi: 10.3389/fpls.2021.807798 (PMC8854266; doi:10.3389/fpls.2021.807798)
Supplement: Supplementary file 1 [file Data_Sheet_1.zip › New folder/Supplementary Figure 7.PPTX]

## Slide 1
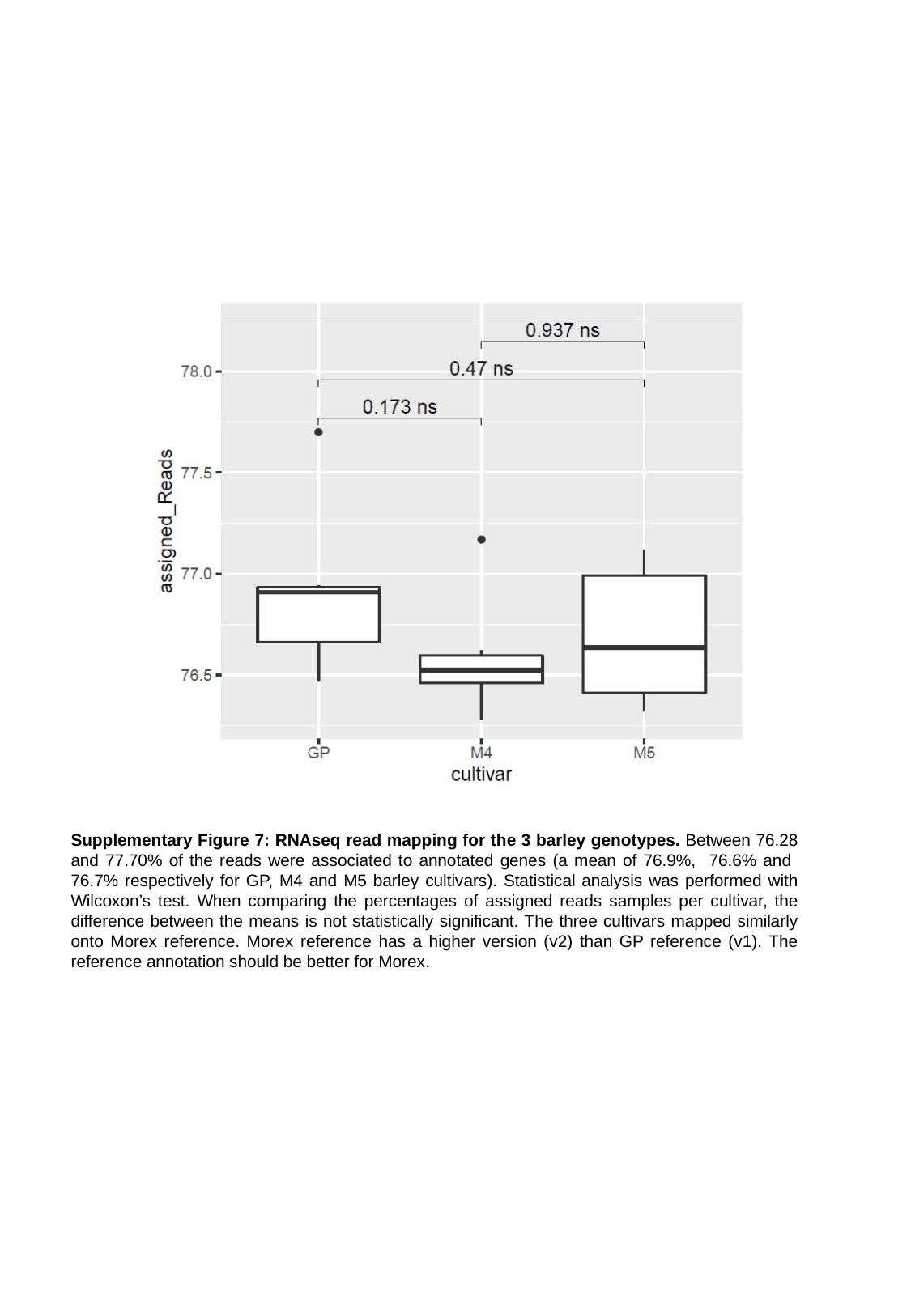

Supplementary Figure 7: RNAseq read mapping for the 3 barley genotypes. Between 76.28 and 77.70% of the reads were associated to annotated genes (a mean of 76.9%, 76.6% and 76.7% respectively for GP, M4 and M5 barley cultivars). Statistical analysis was performed with Wilcoxon’s test. When comparing the percentages of assigned reads samples per cultivar, the difference between the means is not statistically significant. The three cultivars mapped similarly onto Morex reference. Morex reference has a higher version (v2) than GP reference (v1). The reference annotation should be better for Morex.
