# Supplementary material for: Genotypic Variation of Nitrogen Use Efficiency and Amino Acid Metabolism in Barley
Source: Front Plant Sci. 2022 Feb 4;12:807798. doi: 10.3389/fpls.2021.807798 (PMC8854266; doi:10.3389/fpls.2021.807798)
Supplement: Supplementary file 1 [file Data_Sheet_1.zip › New folder/Supplementary Table 3.DOCX]

**Supplementary Table 3:** Expression level of genes up regulated in both GP, M4 or M5 are shown. When the difference between LN and HN value is significantly different, the FC is written in bold. Log2 of the fold changes in LN vs HN are indicated. Bold indicates statistically significant difference between LN and HN. ns: non significant. nd: not detected.

|  |  |  |  |  |
| --- | --- | --- | --- | --- |
| ID | Annotation Hordeum vulgare genome_R2 | log2Fold Change GP LN/HN | log2Fold Change M4 LN/HN | log2Fold Change M5LN/HN |
| HORVU7Hr1G073050 | NAD(P)H dehydrogenase B2 | **1.20** | **1.14** | 0.43 ns |
| HORVU5Hr1G081500 | Senescence-inducible chloroplast protein, Activation of the chlorophll-degrading pathway during leaf senescence | **1.26** | **0.95** | 0.14 ns |
| HORVU3Hr1G097770 | no data | **1.18** | **1.11** | 0.03 ns |
| HORVU2Hr1G027470 | Cytochrome P450, family 709, subfamily B, polypeptide 3 | **1.81** | **1.30** | 0.60 ns |
| HORVU1Hr1G017700 | Low temperature and salt responsive protein family | **1.44** | **1.06** | 0.28 ns |
| HORVU1Hr1G057880 | Eukaryotic release factor 1-2 | **6.51** | **2.17** | nd |
| HORVU2Hr1G070880 | no data | **1.43** | **1.71** | 0.36 ns |
| HORVU3Hr1G088190 | Heavy metal transport/detoxification superfamily protein | **1.39** | **2.24** | 0.47 ns |
| HORVU3Hr1G011820 | Cytochrome P450, family 71, subfamily A, polypeptide 26 | **3.46** | **1.85** | -0.07 ns |
| HORVU7Hr1G098550 | Nitrate transporter2.10 | **2.56** | **2.72** | 2.61 ns |
| HORVU5Hr1G068060 | Phosphate transporter 3;2 | **1.23** | **1.36** | 0.51 ns |
| HORVU5Hr1G062290 | Jasmonate-zim-domain protein 1 | **2.00** | **2.41** | 0.99 ns |
| HORVU1Hr1G089510 | RAD-like 1 | **2.73** | nd | **5.57** |
| HORVU1Hr1G089490 | RAD-like 1 | **1.79** | - 0.03 ns | **2.99** |
| HORVU1Hr1G089500 | RAD-like 6 | **1.47** | 0.30 ns | **3.17** |
| HORVU5Hr1G078960 | 2 iron, 2 sulfur cluster binding | **1.46** | 0.44 ns | **1.05** |
| HORVU6Hr1G060720 | Cytochrome b561/ferric reductase transmembrane protein family | **1.14** | 0.24 ns | **0.74** |
| HORVU5Hr1G047730 | Ferritin 4 | **1.39** | 0.93 ns | **1.47** |
| HORVU2Hr1G007350 | no data | 0.09 ns | **0.77** | **0.67** |
