# Supplementary material for: Genotypic Variation of Nitrogen Use Efficiency and Amino Acid Metabolism in Barley
Source: Front Plant Sci. 2022 Feb 4;12:807798. doi: 10.3389/fpls.2021.807798 (PMC8854266; doi:10.3389/fpls.2021.807798)
Supplement: Supplementary file 1 [file Data_Sheet_1.zip › New folder/Supplementary Table 4.DOCX]

**Supplementary Table 4 : List of genes down-regulated in at least two barley genotypes under LN compared to HN.** Expression level of genes down regulated in both GP, M4 or M5 are shown. When the difference between LN and HN value is significantly different, the FC is written in bold. Log2 of the fold changes in LN vs HN are indicated. Bold indicates statistically significant difference between LN and HN. ns: non significant. nd: not detected.

| ID | Annotation Hordeum vulgare genome_R2 | log2Fold Change GP LN/HN | log2Fold Change M4 LN/HN | log2Fold Change M5 LN/HN |
| --- | --- | --- | --- | --- |
| HORVU2Hr1G013450 | basic helix-loop-helix (bHLH) DNA-binding superfamily protein | **-3.00** | 0.25 ns | **-3.62** |
| HORVU2Hr1G028530 | basic helix-loop-helix (bHLH) DNA-binding superfamily protein | **-0.76** | 0.18 ns | **-1.31** |
| HORVU3Hr1G050080 | Protein of unknown function (DUF1230) | **-0.86** | 0.23 ns | **-1.11** |
| HORVU3Hr1G059130 | OBP3-responsive gene 1 | **-2.73** | 0.10 ns | **-3.22** |
| HORVU3Hr1G064080 | no data | **-1.86** | -0.24 ns | **-1.93** |
| HORVU3Hr1G078390 | F-box family protein | **-4.18** | nd | **-6.81** |
| HORVU3Hr1G082460 | Major facilitator superfamily protein | **-1.55** | 0.50 ns | **-1.88** |
| HORVU3Hr1G082580 | Major facilitator superfamily protein | **-1.89** | 0.31 ns | **-3.16** |
| HORVU3Hr1G108670 | basic helix-loop-helix (bHLH) DNA-binding superfamily protein | **-5.36** | nd | **-5.52** |
| HORVU3Hr1G108680 | basic helix-loop-helix (bHLH) DNA-binding superfamily protein | **-3.94** | -0.21 ns | **-5.15** |
| HORVU4Hr1G009380 | no data | **-2.32** | -0.02 ns | **-3.08** |
| HORVU4Hr1G011250 | no data | **-0.97** | 0.24 ns | **-1.95** |
| HORVU4Hr1G022630 | SOS3-interacting protein 1 | **-1.17** | -1.03 ns | **-1.96** |
| HORVU4Hr1G083400 | beta-hydroxylase 1 | **-1.66** | 0.30 ns | **-1.75** |
| HORVU5Hr1G099020 | Leucine carboxyl methyltransferase | **-0.74** | 0.17 ns | **-0.96** |
| HORVU6Hr1G017920 | chloride channel B | **-0.72** | -0.64 ns | **-1.21** |
| HORVU7Hr1G080510 | phosphoenolpyruvate carboxylase 3 | **-0.97** | -0.66 ns | **-1.11** |
| HORVU7Hr1G108790 | ureide permease 1 | **-1.81** | -1.67 ns | **-2.37** |
